# Essential role of proline synthesis and the one-carbon metabolism pathways for systemic virulence of *Streptococcus pneumoniae*

**DOI:** 10.1128/mbio.01758-24

**Published:** 2024-10-18

**Authors:** Elisa Ramos-Sevillano, Giuseppe Ercoli, Modupeh Betts, José Afonso Guerra-Assunção, Amy Iverson, Matthew Frank, Frederick Partridge, Stephanie W. Lo, Vitor E. Fernandes, Fauzy Nasher, Emma Wall, Brendan Wren, Stephen B. Gordon, Daniela M. Ferreira, Rob Heyderman, Jason Rosch, Jeremy S. Brown

**Affiliations:** 1 Centre for Inflammation and Tissue Repair, UCL Respiratory, Division of Medicine, University College London, Rayne Institute, London, United Kingdom; 2 Research Department of Infection, Division of Infection and Immunity, University College London, Rayne Institute, London, United Kingdom; 3 Great Ormond Street Institute of Child Health, University College London (UCL), London, United Kingdom; 4 Department of Host-Microbe Interactions, St. Jude Children’s Research Hospital, Memphis, Tennessee, USA; 5 School of Life Sciences, University of Westminster, London, United Kingdom; 6 Parasites and Microbes, Wellcome Sanger Institute, Hinxton, United Kingdom; 7 Milner Centre for Evolution, Department of Life Sciences, University of Bath, Bath, United Kingdom; 8 Faculdade de Medicina e Ciências Biomédicas and ABC-RI. Faro, Faro, Portugal; 9 Faculty of Infectious and Tropical Diseases, London School of Hygiene and Tropical Medicine, London, United Kingdom; 10 Malawi-Liverpool-Wellcome Trust Clinical Research Programme Blantyre, Blantyre, Malawi; 11 Liverpool School of Tropical Medicine, Liverpool, United Kingdom; Mississippi State University, Mississippi State, Mississippi, USA

**Keywords:** *Streptococcus pneumoniae*, proline synthesis, formate-tetrahydrofolate ligase, stringent response, virulence

## Abstract

Virulence screens have indicated potential roles during *Streptococcus pneumoniae* infection for the one-carbon metabolism pathway component Fhs and proline synthesis mediated by ProABC. To define how these metabolic pathways affect *S. pneumoniae* virulence, we have investigated the phenotypes, transcription, and metabolic profiles of Δ*fhs* and Δ*proABC* mutants. *S. pneumoniae* capsular serotype 6B BHN418 Δ*fhs* and Δ*proABC* mutant strains had strongly reduced virulence in mouse sepsis and pneumonia models but could colonize the nasopharynx. Both mutant strains grew normally in complete media but had markedly impaired growth in chemically defined medium, human serum, and human cerebrospinal fluid. The BHN418 Δ*proABC* strain also had impaired growth under conditions of osmotic and oxidative stress. The virulence role of *proABC* was strain specific, as the D39 Δ*proABC* strain could still cause septicemia and grow in serum. Compared to culture in broth, in serum, the BHN418 Δ*fhs* and Δ*proABC* strains showed considerable derangement in global gene transcription that affected multiple but different metabolic pathways for each mutant strain. Metabolic data suggested that Δ*fhs* had an impaired stringent response, and when cultured in sera, BHN418 Δ*fhs* and Δ*proABC* were under increased oxidative stress and had altered lipid profiles. Loss of *proABC* also affected carbohydrate metabolism and the accumulation of peptidoglycan synthesis precursors in the BHN418 but not the D39 background, linking this phenotype to the conditional virulence phenotype. These data identify the *S. pneumoniae* metabolic functions affected by *S. pneumoniae* one-carbon metabolism and proline biosynthesis, and the role of these genetic loci for establishing systemic infection.

**IMPORTANCE:**

Rapid adaptation to grow within the physiological conditions found in the host environment is an essential but poorly understood virulence requirement for systemic pathogens such as *Streptococcus pneumoniae*. We have now demonstrated an essential role for the one-carbon metabolism pathway and a conditional role depending on strain background for proline biosynthesis for *S. pneumoniae* growth in serum or cerebrospinal fluid, and therefore for systemic virulence. RNAseq and metabolomic data demonstrated that the loss of one-carbon metabolism or proline biosynthesis has profound but differing effects on *S. pneumoniae* metabolism in human serum, identifying the metabolic processes dependent on each pathway during systemic infection. These data provide a more detailed understanding of the adaptations required by systemic bacterial pathogens in order to cause infection and demonstrate that the requirement for some of these adaptations varies between strains from the same species and could therefore underpin strain variations in virulence potential.

## INTRODUCTION

*Streptococcus pneumoniae* is a common upper respiratory tract commensal but frequently causes invasive infections responsible for approaching a million deaths a year in children (1–3). *S. pneumoniae* has multiple virulence factors (4), including the polysaccharide capsule required for immune evasion (5) and surface proteins also involved in immune evasion as well as adhesion to host cells (6–9). Another essential requirement for virulence is bacterial replication under host physiological conditions (10), and growth in serum differentiates *S. pneumoniae* from the less virulent streptococci (11). Host physiological conditions include a temperature of 37°C, a pH of 7.4, serum osmolality of around 285 mmol/kg, and restricted availability of multiple cations and micronutrients needed for bacterial replication (12, 13). As a consequence, the virulence of *S. pneumoniae* is dependent on cation, polyamine, and amino acid transporters (14–19); effective osmoregulation (18, 20); and synthesis of nutrients with limited availability in the host (21–23). However, our understanding of the *S. pneumoniae* factors required to replicate under physiological conditions remains incomplete.

We analyzed published transcriptome and transposon screen data to identify metabolic pathways involved during infection but yet to be characterized in detail (24–26). Two loci of interest were identified, the *proABC* (SP_0931–33) operon and *fhs* (SP_1229). ProA (Sp_0932) is a γ-glutamyl phosphate reductase, ProB (Sp_0931) a γ-glutamyl kinase, and ProC (Sp_0933) a pyrroline-5-carboxylate reductase responsible for proline synthesis from glutamate (27). Proline protects bacteria against osmostress (28–30), and proline synthesis or transport is important for *Salmonella* Typhimurium and *Mycobacterium tuberculosis* virulence (31, 32). Mutation of *proABC* operon reduced *S. pneumoniae* virulence in mice (24, 33, 34). *fhs* is predicted to encode a formate-tetrahydrofolate ligase that catalyzes the formation of 10-formyl-tetrahydrofolate from folate (as tetrahydrofolate [THF]) and formate. Fhs is part of the one-carbon metabolism pathway which provides cofactors for the synthesis of multiple products. THF donates carbon for the synthesis of amino acids and purines (35, 36), and may contribute to the synthesis of alarmones guanosine-pentaphosphate and -tetraphosphate [(p)ppGpp] that initiate the bacterial stringent response required for adaptation to nutritional and physiological stress (37). THF synthesis in most bacteria is catalyzed by FolD, but a minority of bacteria including *S. pneumoniae* use Fhs (38–41). The one-carbon metabolism pathway could be important for multiple metabolic pathways involved in adaptation to host physiological conditions, and *S. pneumoniae* increases *fhs* expression in media containing low levels of methionine and during mouse meningitis (26, 35). *S. pneumoniae fhs* is described as an essential gene for some strains (37). Mutation of *fhs* reduced *S. pneumoniae* virulence in mouse models of pneumonia or meningitis (24, 26), but its role during infection has not been investigated and could be relevant for other bacterial pathogens that contain *fhs* (41).

Previously we have used *S. pneumoniae* Δ*fhs* and Δ*proABC* strains as live-attenuated *S. pneumoniae* vaccines, demonstrating their potential clinical utility (42, 43). In this study, we have characterized *S. pneumoniae* ∆*proABC* and ∆*fhs* strain phenotypes in detail to determine the roles of proline synthesis and the one-carbon metabolism pathway during disease pathogenesis.

## RESULTS

### Bioinformatic analysis of *fhs* and *proABC*

Analyzing 20,924 pneumococcal genomes demonstrated that the *fhs* and *proABC* genes were highly conserved; all four genes were present in almost all genomes. The exceptions were *proA* and *proC,* which were absent in one serotype 6A strain (GPS_NP_6691). Mean nucleotide similarity across *S. pneumoniae* strains was 99.4%, 98.6%, 96.2%, and 99.9%, respectively, for *proB*, *proA*, *proC*, and *fhs*. The amino acid identity of *S. pneumoniae* TIGR4 ProA, ProB, and ProC predicted proteins was 48%, 42%, and 28% to *Bacillus subtilis* (strain 168) and 46%, 38%, and 40% to *Escherichia coli* (strain K12) ProA, ProB, and ProC (44). The predicted amino acid sequence of *S. pneumoniae* Fhs contains the described active sites, including the ATP-binding domain (PTPAGEGKXT, X is S or T), a glycine-rich nucleotide binding consensus sequence, and folate (Trp412, Phe 385), para-aminobenzoic acid (Pro385, Leu408), or THF (95–103 EPSLGPX_2_G, aspartate at residue 29) binding residues (36, 45–47). PSI-blast based secondary structure prediction (PSIPRED) analysis (48) indicated that Fhs is intracellular. Mutants containing complete deletion of *proABC* or *fhs* were constructed in the serotype 6B strain BHN418 using overlap extension PCR and transferred to the capsular serotype 2 D39 strain using transformation with genomic DNA (Fig. S1). A ∆*fhs + fhs* 6B serotype complemented mutant was constructed by insertion of *fhs* into a neutral genome site using the integration vector pPEPY (49). The Δ*proABC* strain was not genetically complemented as the *in vitro* phenotype was linked to proline directly using growth supplementation (see below).

### ∆*proAB*C and ∆*fhs* strain *in vivo* phenotypes

**Fig 1:**
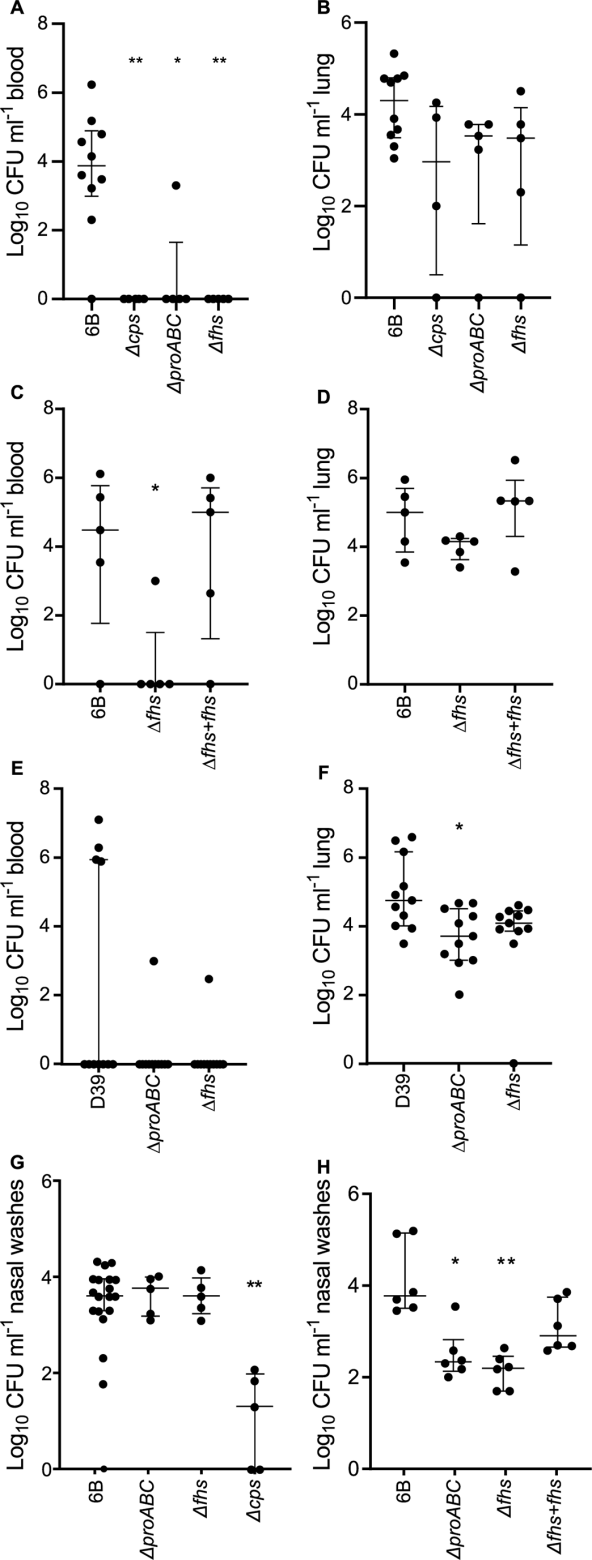
Virulence of the Δ*fhs* and Δ*proABC* mutant strains in pneumonia and colonization models. Log_10_ mL^−1^ bacteria CFU recovered from blood (**A, C, E**) and lung (**B, D, F**) of 5-week-old CD-1 mice 18 hours post-intranasal inoculation with 1 × 10^7^ CFU of the wild-type 6B or D39 and mutant strains ∆*proABC* and ∆*fhs*. Each symbol represents CFU data from a single mouse, horizontal bars represent median values, error bars represent interquartile range, and asterisks represent statistical significance compared to the wild-type strain (Kruskal-Wallis with Dunn’s post hoc test to identify significant differences between groups, *P < 0.05; **P < 0.01). (**G and H**) Colonization model; CFU in nasal washes of CD1 mice 7 (**G**) or 12 days (**H**) post-colonization with 1 × 10^7^  CFU of wild-type 6B or single-mutant *S. pneumoniae* strains. The lower limit of detection reported was 50 CFU mL^−1^; therefore, any values below this threshold are represented as zero.

**Fig 2:**
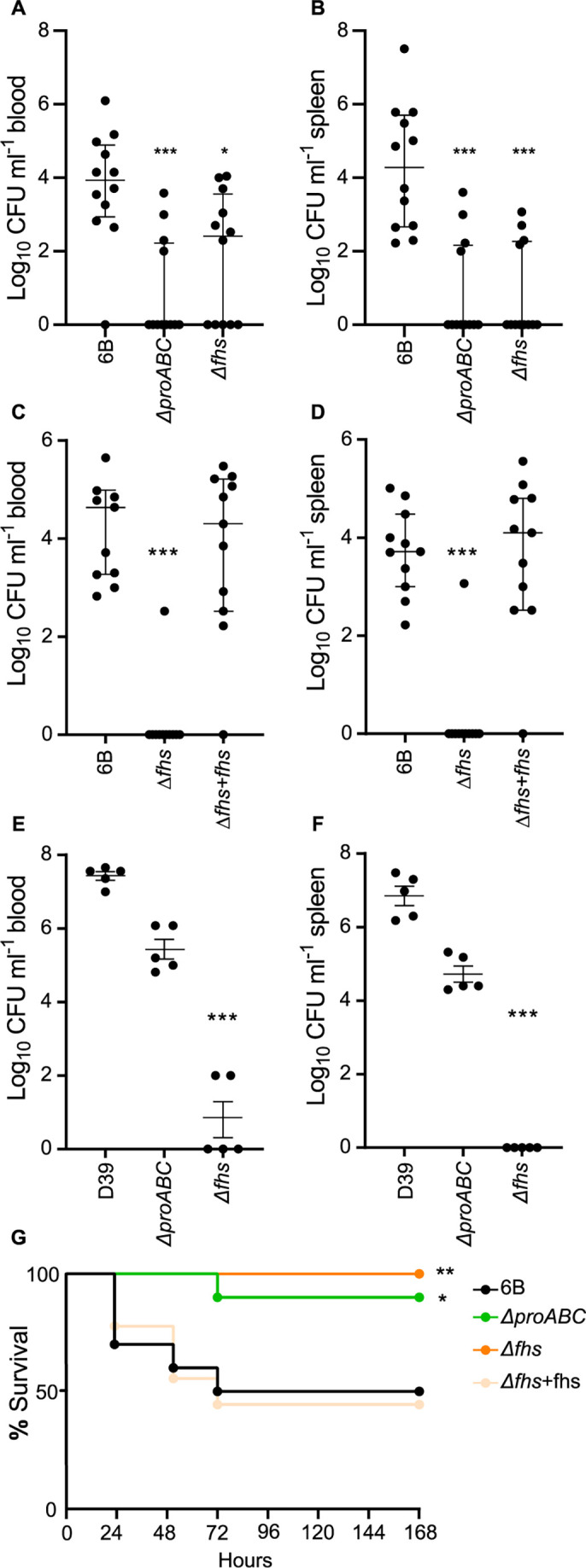
Virulence of the Δ*fhs* and Δ*proABC* mutant strains in a sepsis model and survival analysis of CD-1 mice during pneumococcal pneumonia. Log_10_ mL^−1^ bacteria CFU recovered from blood (**A, C, E**) and spleen (**B, D, F**) of 5-week-old CD-1 mice 24 hours post-intraperitoneal inoculation with 5 × 10^6^ CFU of the wild-type (6B or D39) or mutant strains ∆*proABC*, ∆*fhs,* and the *fhs* complemented mutant strain ∆*fhs + fhs*. Each symbol represents CFU data from a single mouse, horizontal bars represent median values, error bars represent interquartile range, and asterisks represent statistical significance compared to the wild-type strain (Kruskal-Wallis with Dunn’s post hoc test to identify significant differences between groups, *P < 0.05; **P < 0.01; *** *P* < 0.001). (**G**) Survival of 5-week-old CD-1 mice (*n* = 10) infected via intranasal inoculation with 1 × 10^7^ CFU of the wild-type 6B or mutant strains monitored over a 7-day period. Survival curves were compared using the log rank (Mantel-Cox) test (*P < 0.05; **P < 0.01). The lower limit of detection reported was 50 CFU mL^−1^; therefore, any values below this threshold are represented as zero.

The BHN418 Δ*proABC* and Δ*fhs* strains had similar invasive infection phenotypes to Δ*cps*, failing to disseminate from the lungs to the blood (Fig. 1A) and with non-significant reductions in lung CFU in a pneumonia model (Fig. 1B) and showing large reductions in blood or spleen CFU in the sepsis model (Fig. 2A and B). Genetic complementation of BHN418 Δ*fhs* with *fhs* restored virulence in both pneumonia and sepsis models (Fig. 1C and D; Fig. 2C and D), confirming the virulence defect was due to deletion of *fhs*. The D39 Δ*fhs* had a similar virulence phenotype to BHN418 Δ*fhs* in pneumonia (Fig. 1E and F) and sepsis models (Fig. 2E and F), and D39 Δ*proABC* strain had a similar phenotype to BHN418 Δ*proABC* in the pneumonia model (Fig. 1E and F). However, in the sepsis model, the D39 Δ*proABC* strain remained partially virulent with statistically non-significant reductions in blood and spleen CFU (Fig. 2E and F). In contrast to sepsis and pneumonia models and unlike *Δcps*, the BHN418 Δ*proABC* and Δ*fhs* maintained nasopharyngeal colonization at similar levels to wild type at 7 days (Fig. 1G), and 12 days post-colonization still colonized the nasopharynx, although with reduced nasal wash CFU compared to wild type (Fig. 1H). To confirm the differences in target organ CFU-altered disease lethality, pneumonia development was monitored for 7 days after infection with BHN418 *ΔproABC* or Δ*fhs* strains. Furthermore, 50% of the mice inoculated with wild-type 6B or the complemented ∆*fhs* mutant developed fatal infection (Fig. 2G), while 90% and 100% of mice infected with ∆*proABC* or Δ*fhs,* respectively, survived. These data demonstrate that the loss of *fhs* has a profound effect on systemic virulence in both 6B and D39 backgrounds, whereas the effects on virulence of loss of *proABC* were partially strain dependent.

### *S. pneumoniae fhs* and *proABC* were not required for immune evasion

Confocal microscopy provided no evidence that loss of *proABC* or Δ*fhs* altered cell morphology or capsule thickness (Fig. S2A). Neither strain showed increased recognition by complement or antibody or reduced resistance to killing by human neutrophils (Fig. S2B through F). Furthermore, in a nematode infection model that reflects host toxicity caused by *S. pneumoniae* (50, 51), the Δ*fhs* mutant strains killed *Caenorhabditis elegans* as rapidly as the wild type (Fig. S2G and H). The Δ*proABC* mutant showed some delay in killing, with 100% of the worms killed only after 24 hours (Fig. S2H). Overall, these data indicate the reduced virulence of the Δ*proABC* and *Δfhs* mutant strains was not related to increased susceptibility to immune effectors.

### Growth of ∆*proABC* and ∆*fhs* in media and under stress conditions

**Fig 3:**
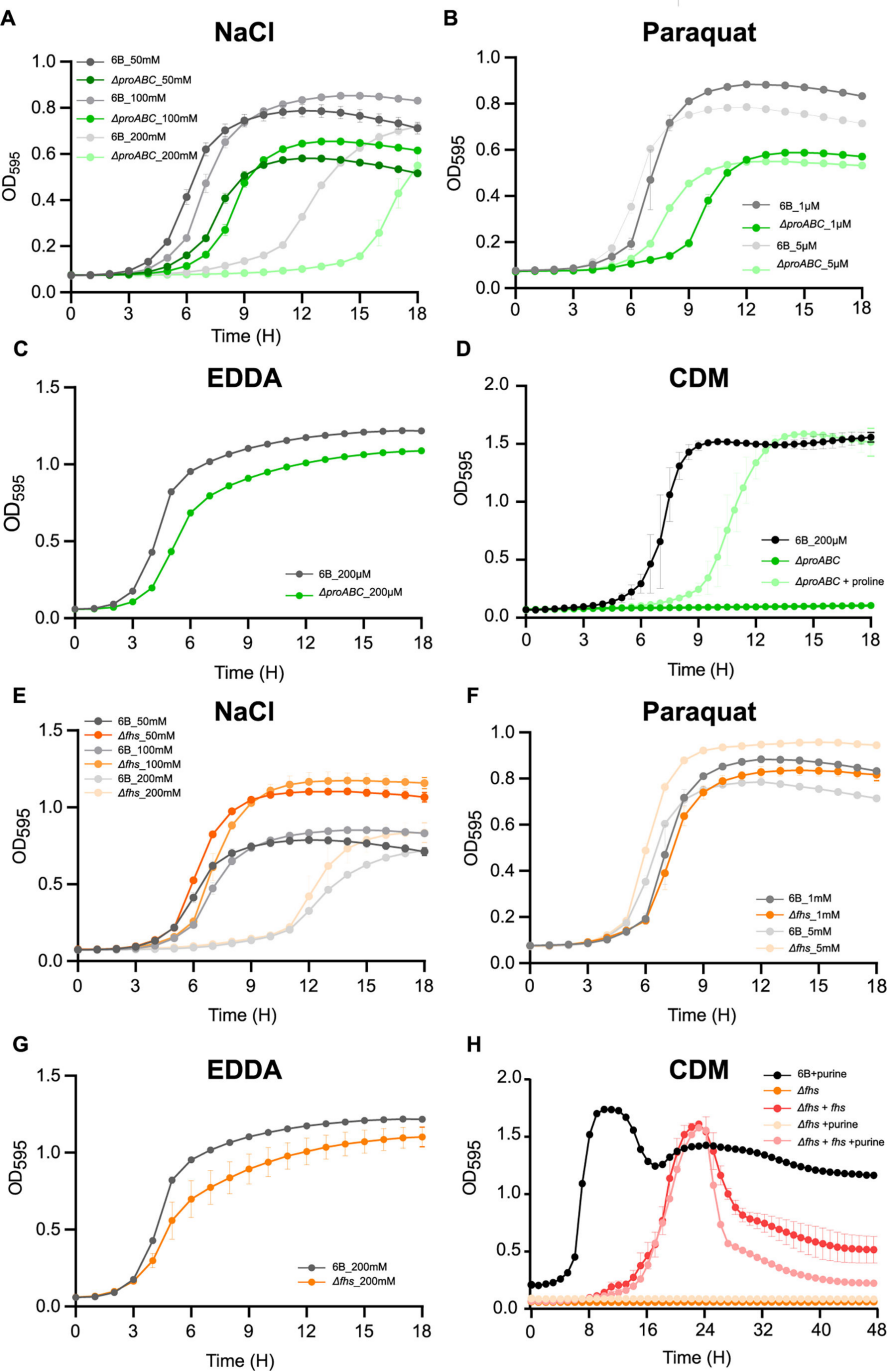
Growth characterization of the Δ*proABC* and Δ*fhs* mutant strains in stress media. Growth of wild-type 6B and Δ*proABC* strains in THY supplemented with (**A**) 50, 100, and 200 mM of NaCl, (**B**) 1 and 5 mM of paraquat, or (**C**) 200 μM of ethylenediamine-N,N′-diacetic acid (EDDA), or (**D**) in CDM media with and without proline supplementation (1 mg mL^−1^). Growth of wild-type 6B and Δ*fhs* strains in THY broth supplemented with (**E**) 50, 100, and 200 mM of NaCl, (**F**) 1 and 5 mM of paraquat, or (**G**) 200 µM of EDDA, or (**H**) in CDM media with or without purine supplementation (1 mg mL^−1^). Growth in all conditions was assessed at 37°C and 5% CO_2_ every 30 min for a period of 24 hours by using a plate reader and measuring OD_595_.

In rich media (Todd-Hewitt broth [THY]), BHN418 ∆*proABC* and ∆*fhs* had identical growth to wild type. Induction of osmotic or oxidative stress by addition of NaCl or paraquat impaired growth of the ∆*proABC* strain (Fig. 3A and B) (44, 52, 53) but did not consistently affect ∆*fhs* growth (Fig. 3E and F). Cation depletion slightly impaired the growth of both ∆*proABC* and ∆*fhs* (Fig. 3C and G). Under conditions with restricted nutrient availability (growth in chemically defined medium, CDM), both ∆*proABC* and ∆*fhs* had severe growth defects compared to wild type (Fig. 3D and H). ∆*proABC* growth in CDM was restored by adding 1 mg mL^−1^ proline (Fig. 3D) but not by proline-containing peptides imported by AliA and AliB or an eight-proline residue oligopeptide (54) (Fig. S3A through C), indicating environmental proline compensated for loss of proline synthesis through proline-specific rather than oligopeptide transporters. Despite the probable role of Fhs in purine synthesis, the addition of purine, adenine, formate, or glycine (known to compensate for poor growth of *E. coli* Δ*folD/p-fhs*) (39) did not restore ∆*fhs* growth in CDM (Fig. 3H, data not shown).

### Poor growth of Δ*fhs* and Δ*proABC* in physiological fluids

**Fig 4:**
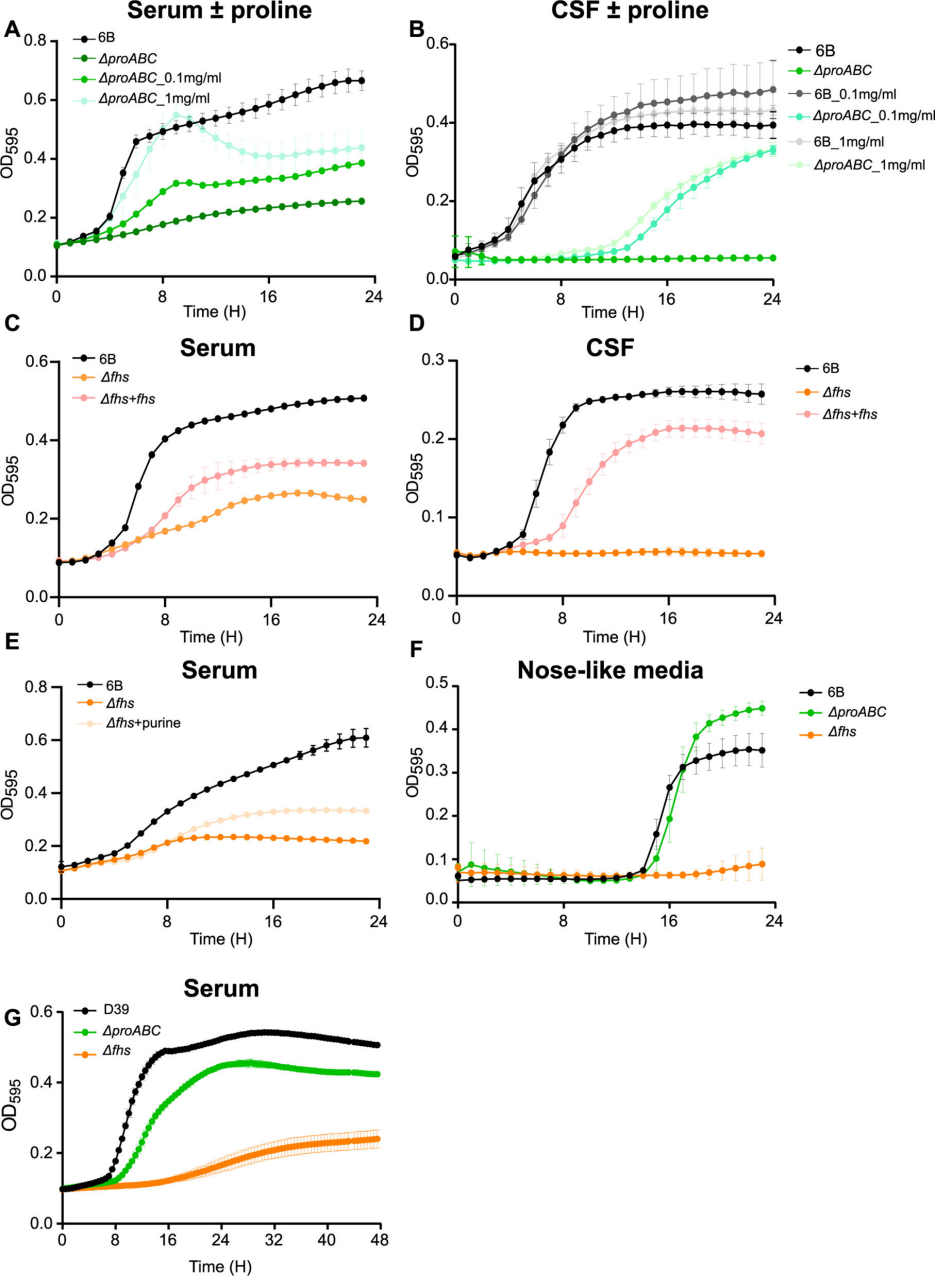
Growth characterization of ∆*proABC* and ∆*fhs* mutant strains in biological fluids. Growth of wild-type 6B and Δ*proABC* mutant strains in (**A**) human serum or (**B**) human cerebrospinal fluid with or without proline supplementation (0.1 or 1 mg mL^−1^). Growth of wild-type 6B, Δ*fhs,* and Δ*fhs* + *fhs* in (**C**) human serum, (**D**) CSF, or (**E**) human serum supplemented with purine 1 mg mL^−1^. (**F**) Growth of wild-type 6B and mutant strains ∆*proABC* and Δ*fhs* in nose-like media (main carbon source N-acetylglucosamine). (**G**) Growth of wild-type D39 and Δ*proABC* mutant strains in human serum. Growth in all conditions was assessed at 37°C and 5% CO_2_ every 30 min for a period of 24 hours by using a plate reader and measuring OD_595_.

**Fig 5:**
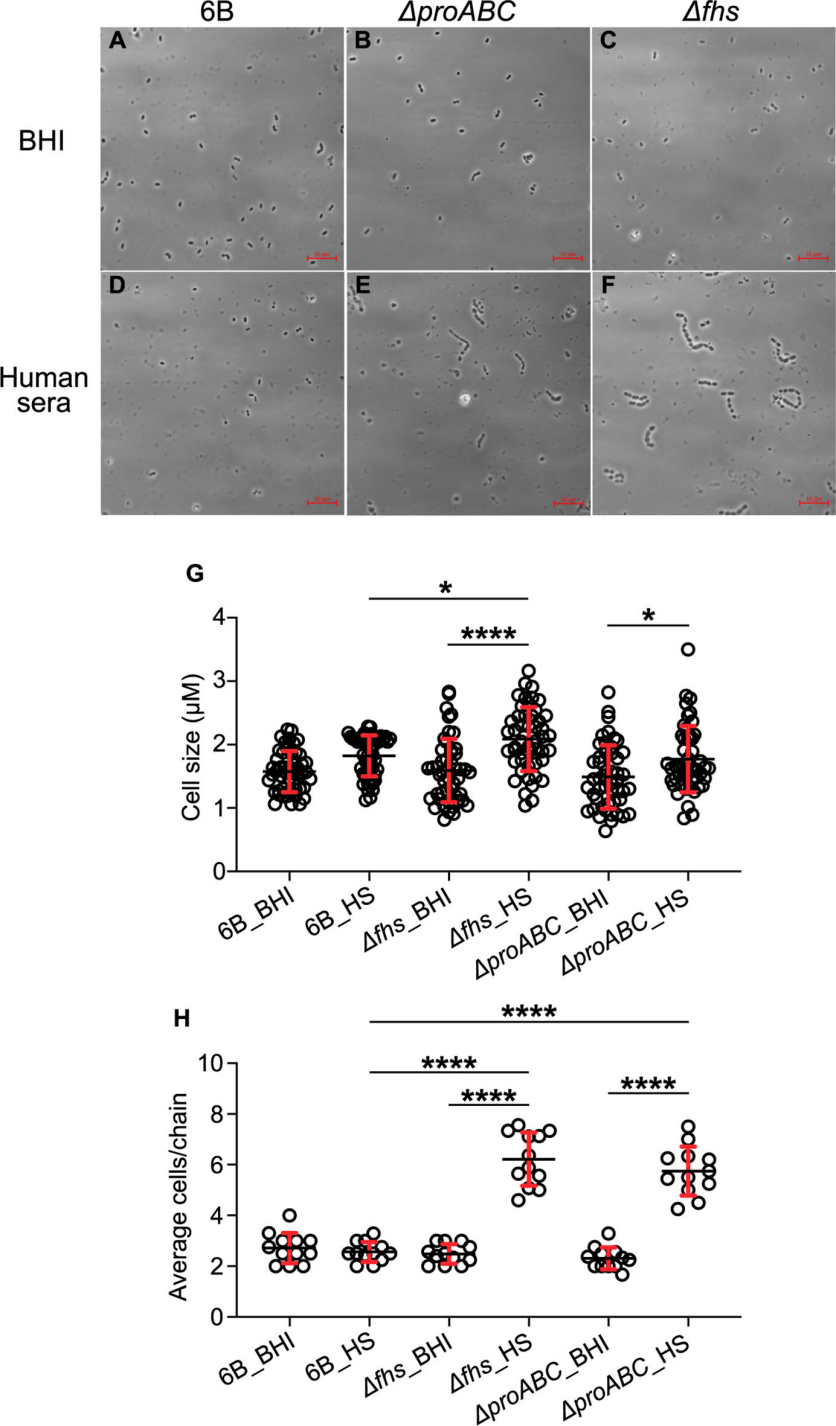
Light microscopy of wild-type 6B and ∆*proABC* and ∆*fhs* mutant strains. Bacteria were incubated in either (**A, B, C**) BHI media or (**D, E, F**) human sera (HS) for a period of 3 hours. The scale bar (bottom right) represents 10 µm. (**G**) Cell size measured from pole to pole in micrometers; diplococci without a clear septum were considered as a single cell. Fifty cells were counted in total for each condition from three independent biological experiments, using Fiji imageJ to measure cell sizes. (**H**) Average chain length. Each circle symbol represents a single chain measurement result, and error bars represent standard deviations. Differences were analyzed using two-way ANOVA, and multiple comparison of columns means (*P = 0.0332; **P = 0.0021; ***P = 0.0002; ****P < 0.0001).

The above experiments suggested poor growth in host physiological conditions could cause the reduced virulence of Δ*fhs* and Δ*proABC*. Hence, their growth was compared to wild type in *ex vivo* 100% human sera or cerebrospinal fluid (CSF). The BHN418 Δ*proABC* mutant was markedly attenuated in growth in sera and CSF (Fig. 4A and B), with growth improved by proline supplementation (Fig. 4A and B). Δ*fhs* also had markedly impaired growth in sera and CSF, which was partially restored for the Δ*fhs + fhs* complemented strain (Fig. 4C and D) or (in sera) by supplementation with purine (Fig. 4E). In a laboratory medium that mimics fluid nasal (55), only the Δ*fhs* mutant had reduced growth compared to wild type (Fig. 4F). When incubated in serum, both mutant strains showed increased chain formation and variable bacterial cell sizes compared to the wild type (Fig. 5). To assess the potential effects of strain background, the growth of D39 ∆*fhs* and Δ*proABC* in serum was investigated. Similar to BHN418 ∆*fhs*, D39 ∆*fhs* had severely impaired growth in serum (Fig. 4G). In contrast, serum could sustain growth of D39 Δ*proABC* (although still impaired compared to wild type), a result compatible with this strain’s maintained ability to cause septicemia in mice. Overall, these data link impaired systemic virulence of the BHN418 and D39 ∆*fhs* and BHN418 Δ*proABC* strains to poor replication in serum and a strain-dependent role for proline synthesis during *S. pneumoniae* pathogenesis.

### RNAseq in THY

**Fig 6:**
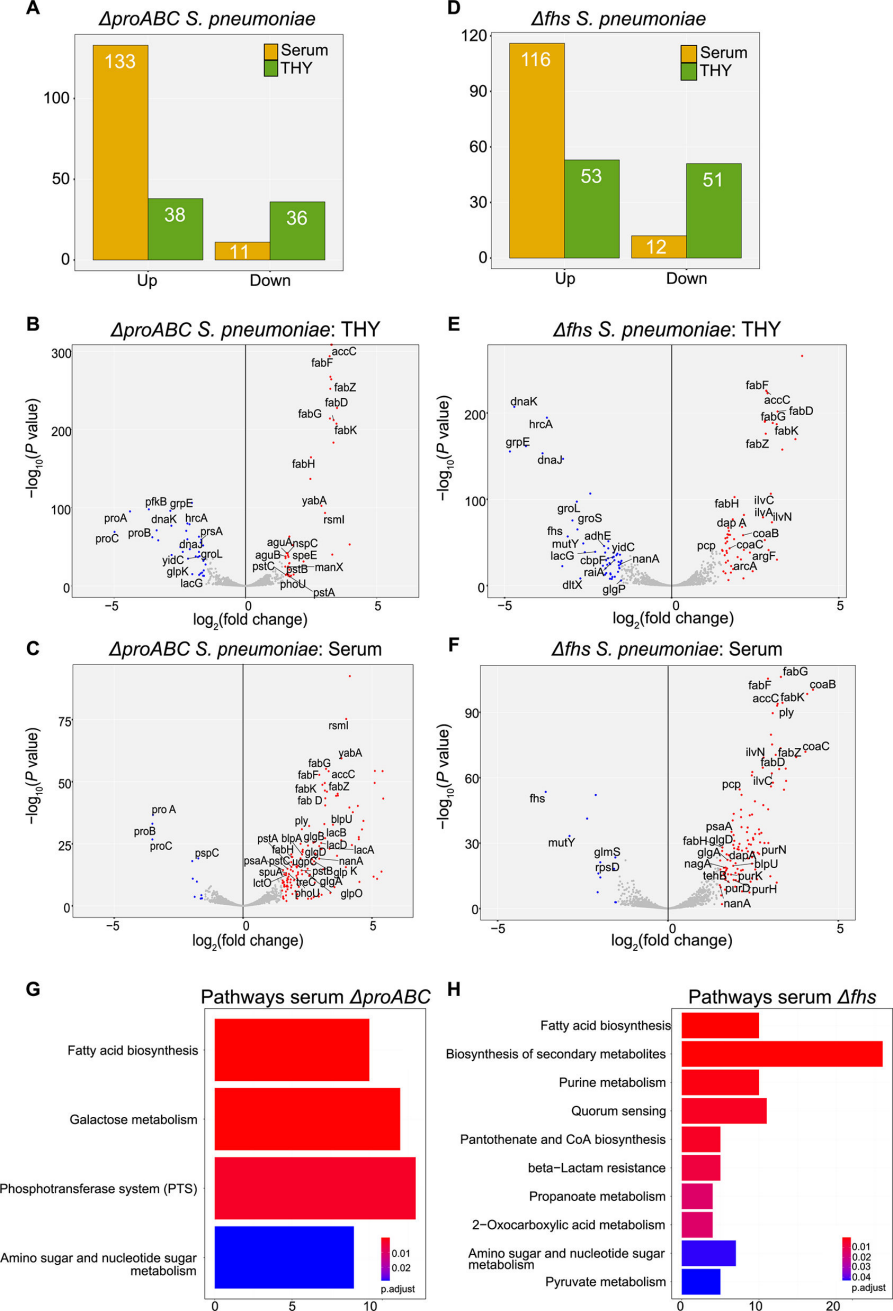
Transcriptome changes for wild-type 6B and ∆*proABC* and ∆*fhs* mutant strains in THY and human serum. (**A**) Total number of differentially expressed genes (DEGs, defined as a log_2_ fold change of >1.5 and false discovery rate [FDR] of <0.05 genes) for ∆*proABC* compared to wild type. Volcano plots showing the individual profiles of DEGs for the ∆*proABC* strain grown in (**B**) THY or (**C**) human serum. (**D**) Total number of DEGs for ∆*fhs* compared to wild type in THY or human serum in THY or human serum. Volcano plots showing the individual profiles of DEGs for the ∆*fhs* strain grown in (**E**) THY or (**F**) human serum. Red dots represent DEGs upregulated by the mutant strains, and blue dots represent downregulated DEGs. Gray-colored dots are transcripts that did not meet logFC and FDR thresholds for DEGs. Short gene names of DEGs are annotated on the plot when available. Data are from three biological replicates. Pathway enrichment analysis for growth of mutants in human serum, compared to wild type. Pathways enriched among the upregulated genes for the (**G**) ∆*proabc* and (**H**) ∆*fhs* mutant strains cultured in serum identified using the KEGG database biological pathway annotations for the *S. pneumoniae* strain SP670-6B and over-representation analysis.

To characterize how *S. pneumoniae* adaptations to growth under physiological conditions were affected by the Δ*proABC* and Δ*fhs* mutations, RNAseq was performed on BHN418 wild-type, Δ*proABC,* and Δ*fhs* 6B incubated in 100% human serum or THY for 60 min. Principal component analysis showed clear separation of serum RNAseq data between strains (Fig. S4), with 90% of the variability from the first two principal components (PC) and 66% from PC1. Selected operons showing changes in expression in serum compared to THY for the wild-type strain are shown in Table 1 and Table S2. In THY, the Δ*proABC* and Δ*fhs* mutant strains showed increased or decreased expression of a similar number of genes compared to the wild-type strain (Fig. 6A and D). Differences in the Δ*proABC* transcriptome in THY to wild type were dominated by genes involved in carbohydrate utilization and biosynthesis (Fig. 6B; Table S3) (56), whereas the ∆*fhs* strain showed upregulation of operons affecting multiple biochemical functions, including amino acid metabolism and synthesis, iron uptake, and other aspects of metabolism (Fig. 6E; Table S3). In THY, both Δ*fhs* and Δ*proABC* upregulated fatty acid synthesis genes and downregulated genes encoding the chaperon proteins GroEL, DnaJK, and the chaperon regulator HrcA (Fig. 6B and E). These data show that despite maintaining growth in THY, the ∆*proABC* and ∆*fhs* strains had significant changes in gene expression likely to reflect bacterial adaptation to the loss of biochemical functions related to each mutation.

**TABLE 1:** Adjacent genes and operons showing differential expression (log_2_ fold change) by the wild-type 6B strain when cultured in sera compared to THY

Strain gene numbers and category			Gene names^*a*^	Function	Log_2_ RNAseq ratio in sera vs THY
6B BHN418	TIGR4	D39			
Amino acid uptake and metabolism					
Spn_00425–29	SP2116-20	SPD1945-49		CAAX amino terminal protease family protein	−2.13, –2.56, −3.14, –3.39, −3.06
Spn_00434–36	SP2125-2126	SPD1954-56		Branched-chain amino acid biosynthetic pathway	−2.79, –2.80, −4.01
Spn_00659–60	SP0112-13	SPD0109-10	*artP1, argG*	Arginino-succinate synthase	−3.11, –3.20
Spn_00839–44	SP0275-80	SPD0255-60	?, *yafQ, _polC_2, pepS,* ? *rsuA1*	Cleavage of amino acid	−3.53, –3.11, −2.77, –3.1, −2.53, –1.6
Spn_01301–05	Spn_0749–53	SP0750-54	*livJ, livH, livM, lptB , livF*	BCAA* ABC transporter	−3.38, –2.26, −2.03, –1.87, −2.19
Spn_01699	SP1159	SPD1023	*XERS*	tyrosine recombinase	−3.48
Sugar uptake and metabolism					
Spn_00150–52	SP1882-84	SPD1662-64	*treC, treP, treR*	Sucrose metabolism	4.55, 4.50, 2.20
Spn_01423–25	SP0875-77	SPD0771-3	*fruR, fruB, fruA*		5.28, 4.99, 4.69
Other metabolism					
Spn_00128	SP1859	SPD_1640	*pnuC*	Nicotinamide mononucleotide transporter	−6.57
Spn_0136–39	SP1869-72	SPD1649-52	*feuB, fepD1, FHUc, yclQ*	Iron transport	1.78, 1.81, 1.77, 1.79
Spn_00311	SP2016	SPD_1826	*nadC*	Nicotinate-nucleotide pyrophosphorylase	−4.44
Spn00603-7, 00609, 00611	SP0044-48, 50, 53	SPD0051-55, 0057, 0059	*purC, purl, purF, purM, purN, purH, purE*	Purine/biotin/coenzyme A synthesis	−2.31, –3.44, −2.68, –2.71, −2.32, –2.26, −1.74
Spn_01501–02	SP0963-64	SPD0851-52	*pyurK, pyrDb*		−4.46, –3.86
Spn_01807–10	SP1275-78	SPD1131-34	*carB, carA, pyrB, pyrR*	Pyrimidine synthesis	−3.50, –3.82, −3.63, –3.40
Spn_00482–85	SP2173-76	SPD_2002–6	*dltD, dltC, dltB, dltA*	Cell wall synthesis	3.18, 2.82, 2.95, 2.76
Other					
Spn_00601–2	SP0042-43	SPD0049-50	*comA, comB*	Competence factor transport proteinS	−1.99, –1.80
Spn_00698	SP0141	SPD_0144	*mutR*	Positive transcriptional regulator of mutA	−4.15
Spn_00914	SP0366	SPD_0334	*aliA*	Oligopeptide ABC transporter	−5.16
Spn_01082	SP0517	SPD0460	*dnaK3*	Molecular chaperone	−4.31
Spn_01631_pulA_2	Sp1118	SPD1002	*pulA2*	Pullulanse	3.04
Spn_02064	SP1161	SPD1025	*lpd*	Dihydrolipoamide dehydrogenase	3.11

^*a*^ "?" represent genes that do not have a gene name.

### Marked disruption of gene expression by the Δ*proABC* and Δ*fhs* strains in serum

When cultured in serum, there was a marked increase in genes showing increased expression compared to wild type for both ∆*proABC* (133 in serum vs 36 in THY) and ∆*fhs* (116 in serum vs 51 in THY) (Fig. 6A and D), demonstrating the mutants underwent major compensatory gene expression changes under infection-related conditions. In serum Δ*proABC,* increased expression of 10 operons involved in sugar uptake and metabolism and 4 operons containing genes of unknown function (Fig. 6C; Table 2; Table S4). In contrast, in sera ∆*fhs,* upregulated operons involved in amino acid uptake or biosynthesis, teichoic acid and coenzyme A biosynthesis, and competence (Fig. 6F; Table 2; Table S4). Genes showing increased expression in serum for both Δ*proABC* and ∆*fhs* included *ply* (encodes pneumolysin), fatty acid and purine biosynthesis operons, and bacteriocin systems. Which pathways were enriched among the upregulated genes were identified using the KEGG biological pathway annotations for *S. pneumoniae* strain SP670-6B and over-representation analysis (57). Δ*proABC* showed significant enrichment for fatty acid biosynthesis, galactose metabolism, PTS systems, and amino acid and sugar metabolism pathways (Fig. 6G). The ∆*fhs* strain showed enriched expression of genes from multiple metabolic pathways, including biosynthesis of secondary metabolites, competence, and purine, pyruvate, propanoate, amino acid, and sugar metabolism (Fig. 6H). To provide a more detailed analysis, expression of all genes within six pathways selected from the above results was analyzed (Fig. S5). In THY, the ∆*proABC* and the ∆*fhs* strains had increased gene expression for two (Fig. S5C and F) and none, respectively, of the six pathways assessed. In contrast, in serum, both mutant strains showed significant increases in gene expression for all six pathways. This result further demonstrates that culture in serum triggered multiple compensatory metabolic responses by ∆*proABC* (Fig. S5A through F) and ∆*fhs* (Fig. S5G through L), which partially differed between the two strains, reflecting the specific roles of *fhs* or *proABC* for *S. pneumoniae* physiology during systemic infection.

**TABLE 2:** Gene operons showing differential expression (log_2_ fold change) between the mutant strains *∆proABC* and *∆fhs,* and the wild-type 6B strain specifically when cultured in human serum (excluding those also showing differences in THY)^*c*^

Strain gene numbers and category			Gene names	Function	log_2_ RNAseq ratio vs wild type in serum	
6B BHN418	TIGR4	D39			Δ*fhs*	Δ*proABC*
Amino acid uptake and metabolism						
Spn_00085–91	SP1811-17	SPD1596-1602	*trpABFCD2GE*	Tryptophan synthesis	ns^*b*^	1.552, 1.940, 1.868, 2.629, 2.800, 2.058, 3.468
Sp_00156–59	SP1887-90	SPD1667-9, 1787	*amiFEDC*	Oligopeptide ABC transporter	2.153, 2.164, 1.953, 1.670	ns
Spn_00659–60	SP0112-13	SPD109-10	*artP1, argG*	Arginino-succinate synthase	2.164, 1.743	ns
Spn_01302–06	SP0750-54	SPD0653-57	*livHMGF,* ?	BCAA^*a*^ ABC transporter	1.803, 1.992, 2.277, 2.262, 1.845	ns
Spn_02005–06	SP1526-27	SPD1354, 1357	*lmrA, aliB*	Oligopeptide ABC transporter	3.335, 2.835	ns
Sugar uptake and metabolism						
Spn_00121–22	SP1852-53	SPD1633-34	*galTK*	Galactose metabolism	ns	2.040, 2.112
Spn_00416–17	SP2109-10	SPD1935-36	*malFG*	Maltodextrin ABC transporter	1.968, 1.510	ns
Spn_00437–39	SP2127-29	SPD1957-59	*tktC*	Transketolase, PTS transporter	ns	1.547, 1.532, 1.554
Spn_00470–76	SP2161-67	SPD1989-95	*manZY, levE1,* ?*, fucUAK*	PTS transporter, fucolose metabolism	ns	1.655, 1.774, 1.838, 2.032, 2.251, 3.135, 3.457
Spn_00617–23	SP0060-64 SP0265-66	SPD0065-71	*bgaC, PTS-EIIB, manZ, PTSII, agaS, mro*	Beta-galactosidase, PTS transporter	ns	6.648, 6.303, 5.690, 5.571, 5.226, 5.001, 3.978
Spn_00816–20	SP0249-53	SPD0233-37	*gmuB, C, hpdB, fsaA, gldA*	PTS transporter	ns	1.915, 2.724, 2.554, 2.110, 1.897
Spn_00879–83	SP0321-25	SPD0293-97	*manX, ugl, levE2, agaC, manZ*	PTS transporter	ns	2.104, 2.576, 2.120, 2.016, 2.035
Spn_01193–97	SP0645-48	SPD0559-62	?, ?*, gatC2,* ?, *lacZ*	PTS transporter, B-galactosidase	ns	5.451, 5.173, 5.105, 4.106, 4.517
Spn_01634–36	SP1122-24	SPD1006-08	*glgCDA*	Glucose metabolism	1.851,1.864,1.692	2.915, 2.755, 2.447
Spn_01728–31	SP1190-93	SPD1050-53	*lacD2B2A*	Tagatose and galactose metabolism	ns	2.391, 2.987, 3.101, 3.097, 3.212
Spn_02152–058	SP1681-85	SPD1493-97	*ycjP4, ugpA, yesO, ptsG, nanE, ugpC*	N-acetylmannosamine ABC transporter	2.042, 2.288, 1.825, 1.679, 1.546	3.042, 3.678, 3.496, 4.381, 4.044
Competence						
Spn_00260–61	SP1980-81	SPD1777-78	*cbf1,* ?	?, Competence-induced protein	ns	1.599, 1.573
Spn_00601–02	SP0042-43	SPD0049-50	*comAB*	CSP ABC transporter permease	1.874, 2.152	ns
Other metabolism						
Spn_00115–16	?, SP1847	?, SPD1628	*xpt, ygfU*	Putative xanthine ABC transporter	3.416, 3.398	2.101, 2.423
Spn_00236–37	SP1956-57	SPD1754-55	?, *ftsE*	Unknown substrate ABC transporter	ns	2.415, 2.086
Spn_00266–67	SP1986-87	*SPD1783-84*	?*, macB*	Unknown substrate ABC transporter	2.024, 2.414	ns
Spn_00494_95	SP2185-86	SPD2012-13	*glpOK*	Glycerol metabolism	ns	2.567, 2.805
Spn_00640–41	SP0090-91	SPD0088-89	*ugpA, ycjP2*	Unknown substrate ABC transporter	ns	1.517,1.620
Spn_00851–52	SP0287-88	SPD026768	*pbuO*	Xanthine/uracil ABC transporter	2.173, 2.580	ns
Spn_01495–96	SP0957	SPD0845	*kpsT*	Unknown substrate ABC transporter	ns	3.604, 3.964
Spn_01799–03	SP1267-71	SPD1123-27	*licC,* ?, *idnD, tarI*	Teichoic acid synthesis	2.442, 2.537, 2.269, 2.256	ns
Spn00965-75	SP0417-27	SPD0380-90	*Fab* operon	Fatty acid synthesis	ns, ns, 3.283, 3.180, 3.206, 2.728, 2.863, 3.002, 3.067, 2.890, 2.931	1.711, 1.570, 3.043, 3.142, 3.142, 2.781, 3.022, 3.107, 3.194, 3.019, 3.066
Miscellaneous						
Spn_01780–81	SP1247-48	SPD1104-05	*ybjI, smc*	Chromosome segregation, ribonuclease	ns	1.772, 1.765
Unknown function						
Spn_00663,65, 67,69	SP0115	SPD0123, 0118	*–*	Hypothetical proteins	ns	2.447, 1.600, 1.585, 1.550
Spn_01232–34	SP0684-86	SPD0596	*–*	Hypothetical proteins	ns	2.998, 1.778, 2.791
Spn_01242–45	SP0703-06	SPD0610-13	*–*	Hypothetical proteins	ns	2.067, 2.262, 1.669, 1.830
Spn_02148–51	SP1677-80	SPD1490, 1492	*–*	Hypothetical proteins	2.395, 2.332, 2.358, 2.418	2.780, 2.777, 3.005, 3.350
Spn_02181–82	SP1707-08	–	*–*	Hypothetical proteins	ns	2.502, 2.488
Purine/biotin/coenzyme A synthesis						
Spn_00603–12	SP0044-54	SPD0051-59	*purCLFMN,* ?, *purHDEK*	Purine synthesis	3.518, 3.857, 3.442, 3.320, 3.039, 2.049, 2.588, 2.265, 2.102, 2.640	1.877, 2.879, 2.601, 2.503, 2.099, 2.055, 1.900, 2.477, 1.877, 2.879
Spn_01765–67	SP1230-31	SPD1088-89	*coaB1B2, panT*	Coenzyme A synthesis	4.255, 4.067, 4.049	ns
Spn_01957–58	SP1470-71	SPD1300-01	*apbE, azr_1*	Thiamine biosynthesis,	ns	1.524, 1.533
Bacteriocins/toxins						
Spn_00213–16	SP1923-26	SPD1726-29	*Ply,* ?, ?, ?, ?	Pneumolysin, unknown	3.128, 3.311, 2.926, 2.590	2.507, 2.647, 2.245, 2.077
Spn_01094–102	SP0529-33	SPD0471-72	*lcnD1D2, lagD2, blpA2,* ?, *blpIN*	Bacteriocin operon	ns, ns, ns, 2.380, ns, 2.814, 2.800	2.024, 1.677, 2.202, 3.702, 3.391, 4.556, 4.650
Spn_01108–11	SP0544-47	SPD0473-75	*blpX, pncO, blpZ,* ?	Bacteriocin operon	1.917, 3.024, 2.397, 2.448	3.826, 4.701, 4.284, 3.871

^*a*^ BCAA, branch-chained amino.

^*b*^ ns, not statistically significant.

^*c*^ "?" and "–" represent genes that do not have a gene name.

### Metabolomic analysis of Δ*proABC* and Δ*fhs*

**Fig 7:**
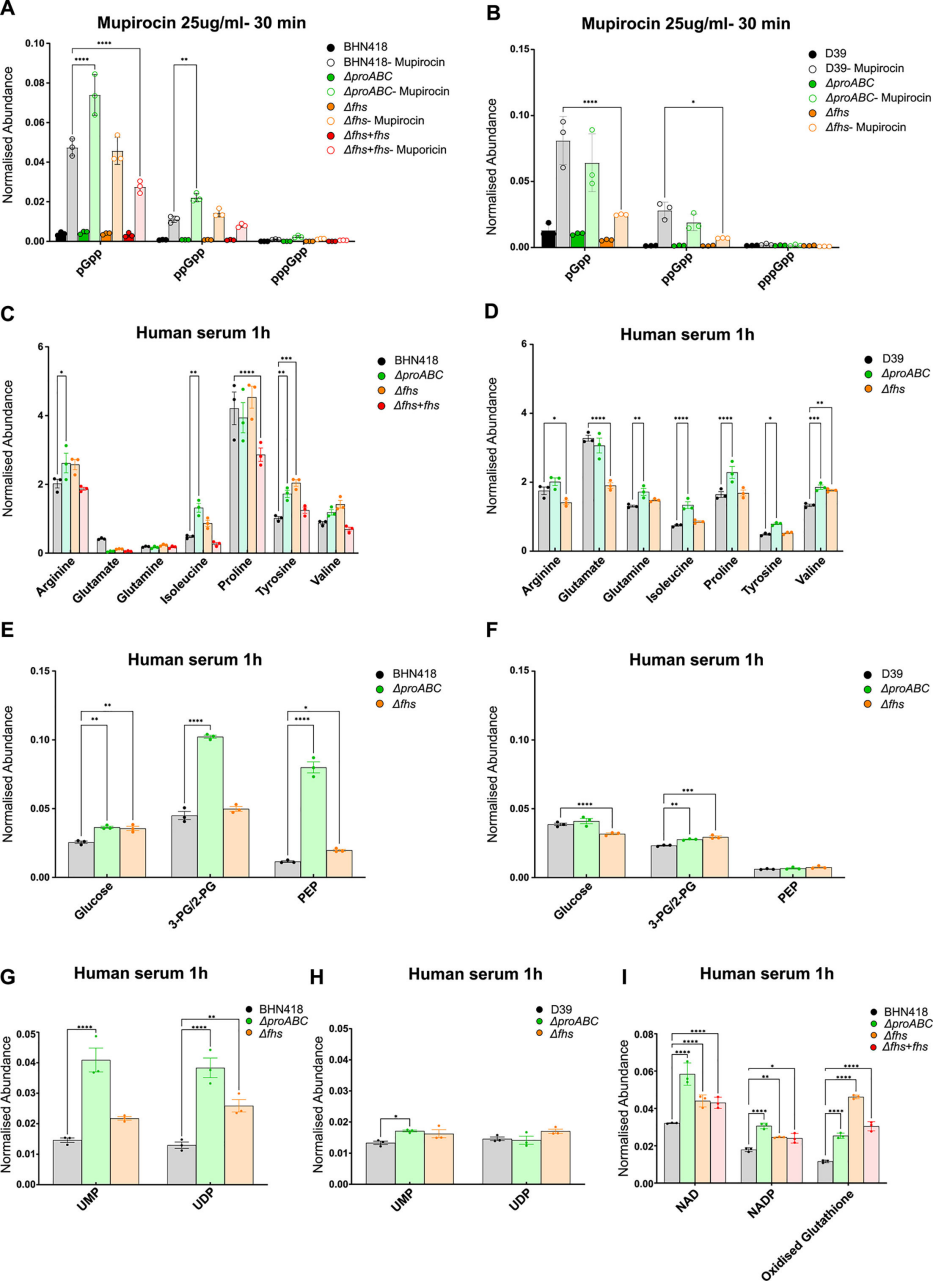
Metabolomic analyses of wild-type strains BHN418 and D39 compared to ∆*proABC*, ∆*fhs,* and ∆*fhs + fhs* mutant strains. Intracellular levels of metabolic components were measured after 1 hour incubation in THY (**A and B**) or human serum (C–I) and represented as relative normalized abundances. Intracellular levels of the alarmones pGpp, ppGpp, and pppGpp in response to the addition of mupirocin in (**A**) BHN418 and (**B**) D39. (**C and D**) Selected intracellular amino acids, (**E and F**) tricarboxylic acid cycle components, (**G and H**) UMP and UDP nucleotides, and (**I**) markers of oxidative stress in BHN418 and D39 wild-type strains. Asterisks indicate significant differences between the wild-type and the mutant strains when assessed using two-way ANOVA (*P  <  0.05; **P  <  0.01; ***P  < 0.001; ****P  <  0.0001).

**Fig 8:**
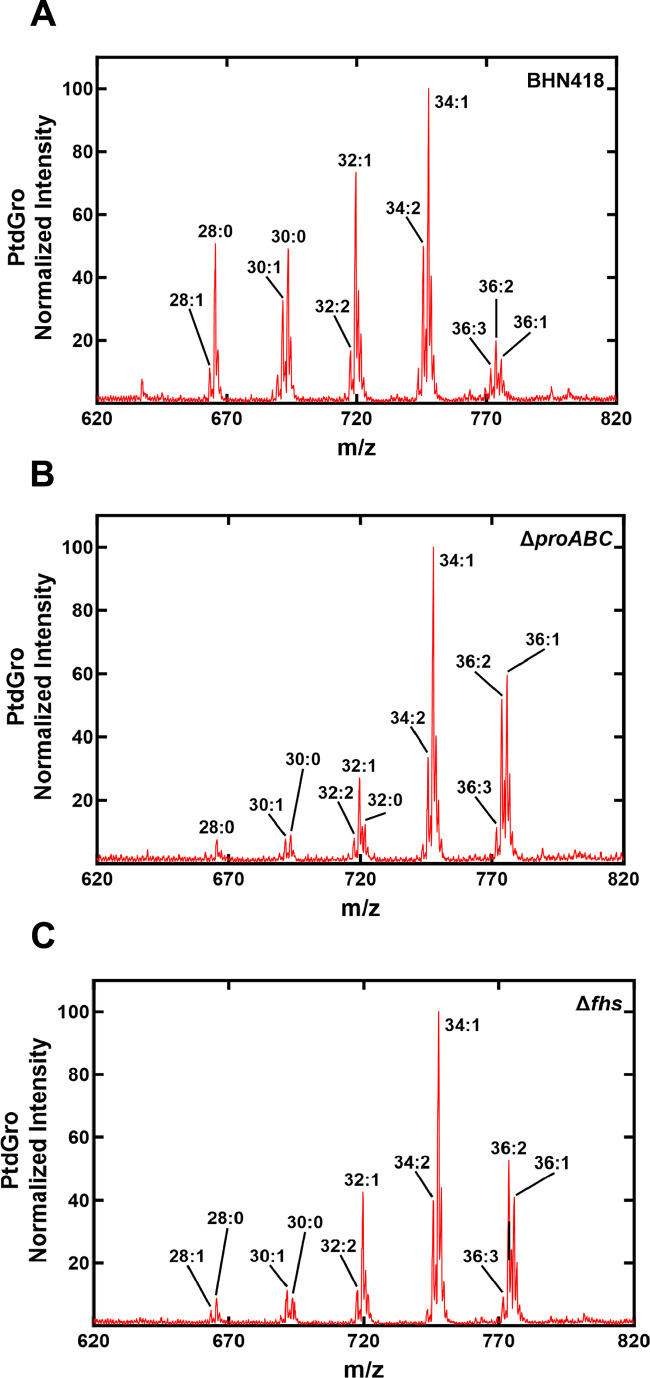
Fatty acid abundance for wild-type BHN418, ∆*proABC,* and ∆*fhs* strains in the presence of serum. Total abundance of saturated and unsaturated acyl chains as determined by LC/MS metabolomic for (**A**) BHN418, (**B**) ∆*proABC*, and (**C**) ∆*fhs* strains, showing an increase in fatty acid chain length (a shift to the right) for the mutant strains compared to wild type.

To further explore the role of ProABC and Fhs for *S. pneumoniae* metabolism and during growth in sera, a metabolomic analysis was performed for BHN418 and D39 wild-type, Δ*proABC* and Δ*fhs* strains incubated in THY or sera. Initially, we assessed the stringent response by incubating bacteria with mupirocin and measuring levels of the alarmones pGpp, ppGpp, and pppGpp. In THY, both the BHN418 and D39 Δ*fhs* had reduced levels of pGpp compared to wild type, indicating a potentially impaired stringent response (Fig. 7A and B). In contrast, the BHN418 Δ*proABC* (but not D39 Δ*proABC*) had increased levels of pGpp and ppGpp, indicating an exaggerated stringent response. Significant artifact effects on alarmone levels prevented measuring the stringent response in serum (data not shown). Unexpectedly, there were only small differences in intracellular concentrations of proline and other amino acids between the corresponding wild type and BHN418 or D39 Δ*proABC* and Δ*fhs* cultured in serum (Fig. 7C and D; Fig. S6). Instead, BHN418 ∆*proABC* (but not the D39 Δ*proABC*) had higher concentrations of intracellular 2- and 3-phosphoglycerate and phosphoenolpyruvate (PEP) (Fig. 7E and F), compatible with impaired metabolism through the Krebs cycle or pentose phosphate pathway and with the RNAseq data, indicating that sugar metabolism was affected by loss of *proABC*. Intracellular phosphorylated uracil nucleotides involved in peptidoglycan synthesis were increased in both the BHN418 ∆*proABC* (UMP, UDP) and Δ*fhs* (UDP) strains but not the D39 ∆*proABC* (Fig. 7G and H), indicating this metabolic effect could be affecting differences in serum growth and morphology phenotypes between BHN418 and D39 ∆*proABC* (Fig. 4 and 5). In sera, both the BHN418 ∆*proABC* and Δ*fhs* had raised intracellular oxidized glutathione, indicating they were under increased oxidative stress (Fig. 7I). Lastly, compatible with upregulation of the fatty acid synthesis operon, there was a significant shift in fatty acid mix for BHN418 ∆*proABC* and Δ*fhs* (Fig. 8) from a mixture of di-saturated and mono- and di-unsaturated phosphatidylglycerol (PtdGro) species with predominant peaks of 28, 30, 32, and 34 total carbons for wild type to mostly mono- and di-unsaturated PtdGro species with an increase in the 36 total carbon peaks and a decrease in 28, 30, and 32 total carbon peaks.

## DISCUSSION

We have investigated *S. pneumoniae fhs* and *proABC,* which are predicted to be important for different key aspects of bacterial metabolism, and shown both the BHN418 Δ*fhs* and Δ*proABC* 6B strains were severely attenuated in virulence in mouse models to a similar level as the unencapsulated mutant. *In vitro* characterization demonstrated poor growth of the BHN418 Δ*fhs* and Δ*proABC* strains in serum or CSF, phenotypes that will largely prevent *S. pneumoniae* from causing septicemia or meningitis, respectively, thereby explaining the loss virulence. Culture under specific stress conditions identified the Δ*proABC* but not Δ*fhs* had increased sensitivity to osmotic and oxidative stress. Furthermore, we demonstrated there were major differences between the Δ*proABC* and Δ*fhs* strains in their RNAseq and metabolomics response to culture in serum, representing different defects in metabolic pathways relevant for growth in serum.

The amino acid proline can be synthesized from glutamate or acquired from the environment (58, 59). The BHN418 ∆*proABC* strain only grew in CDM (contains 0.1 mg/mL of proline) supplemented with 1 mg/mL proline, linking its growth defect to loss of proline synthesis and demonstrating a central role for proline synthesis for *S. pneumoniae* growth that can only be bypassed by high levels of environmental proline. *S. pneumoniae* has no known equivalent to the high-affinity proline transporters of *Bacillus subtilis* (*opuE*) (58, 59) or *S. aureus* (*putP* and *proP*) (60). Proline concentrations in human serum (0.002 mg/mL) are far lower than in CDM (61), explaining why the BHN418 Δ*proABC* mutant was unable to grow in serum or CSF without proline supplementation. Why proline supplementation with 0.1 mg/mL partially restored Δ*proABC* growth in serum, but not CDM, is not clear; possibly, serum and CSF provide some proline from peptide sources or have higher concentrations of other nutrients that compensate for loss of proline. As the metabolomic data are not quantitative, we cannot state what the concentration of intracellular proline was in *S. pneumoniae*. Unexpectedly, the metabolomic data demonstrated that intracellular proline levels were not reduced in Δ*proABC*; potentially, the intracellular proline pool was maintained by restricting proline use for biosynthesis and secondary metabolism, thereby creating significant metabolic stress. We reasoned that genes showing increased expression by Δ*proABC* in serum represent compensatory metabolic pathways activated in response to loss of proline availability and, therefore, the metabolic stress placed on the organism by loss of *proABC*. Unexpectedly, these pathways were dominated by carbohydrate rather than amino acid uptake and metabolism genes, results which were reinforced by the metabolomic data showing significant increases in glycolytic pathway intermediates in BHN418 Δ*proABC*. These results suggest that proline deficiency adversely affects *S. pneumoniae* carbohydrate metabolism during growth in serum or CSF. Proline availability could also affect *S. pneumoniae* growth via its role in osmoregulation (29, 62–65), and the Δ*proABC* mutant was indeed more sensitive to osmotic stress. In addition, loss of proline synthesis could impair synthesis of proline-rich virulence proteins, such as PspC and PspA (66, 67).

Although *fhs* was identified by *S. pneumoniae* virulence screens (24, 26) and is required for *Streptococcus suis* infection (68), the role of Fhs during infection seems to be under-appreciated. *S. pneumoniae* growth in serum or CSF was totally dependent on *fhs*, demonstrating a central role for one-carbon metabolism (37, 41) for *S. pneumoniae* metabolism under physiological conditions. Several metabolic roles have been identified for Fhs in other bacteria, including anaerobic growth (39), purine synthesis (36), and folate homeostasis (41). Exogenous purines partially restored ∆*fhs* growth in serum, and ∆*fhs* upregulated purine pathways in serum. In addition, both D39 and BHN418 ∆*fhs* strains had impaired formation of alarmones in response to mupirocin. These data suggest *S. pneumoniae* purine metabolism and the stringent response are both dependent on Fhs. In addition, the RNAseq and metabolomic data indicated *S. pneumoniae* Fhs has multiple metabolic roles during growth in serum, with loss of *fhs* resulting in increased oxidative stress and altered lipid metabolism. Furthermore, the accumulation of UDP, increased expression of beta-lactam resistance genes, and changes in bacterial morphology in Δ*fhs* indicated potential effects on peptidoglycan synthesis. In combination, these effects severely impaired growth in sera or CSF and rendered the ∆*fhs* strain incapable of systemic virulence.

Despite the severe attenuation of the 6B Δ*fhs* and Δ*proABC* strains during invasive infection, these strains were still able to persist in the nasopharynx, a phenotype we exploited to make live-attenuated *S. pneumoniae* vaccines (42, 43). Why the physiological conditions in the respiratory tract result in reduced dependence on proline synthesis and one-carbon metabolism for *S. pneumoniae* growth is not clear. This could reflect different carbohydrate sources, with the nasopharynx containing several alternative carbohydrates to glucose known to support *S. pneumoniae* growth (glucose) (69) or the more rapid replication by *S. pneumoniae* in blood (increasing from 0 CFU to approximately 10^4^/mL within 24 hours). *S. pneumoniae* essential genes can be divided into universal, core-strain-specific, and accessory essential gene categories (37). *fhs* was described as a core-strain-specific essential gene, but we and others (24, 26) have shown *fhs* is non-essential for growth in rich media but essential for growth in blood, CSF, or CDM, further illustrating that gene essentiality is dependent on growth conditions. Unlike the BHN418 Δ*proABC* strain, the D39 Δ*proABC* strain could replicate in blood *ex vivo* and caused a reduced level of septicemia in the sepsis model, demonstrating that the ProABC role during *S. pneumoniae* invasive infection is strain dependent. The effects of Δ*proABC* mutation in BHN418 on carbohydrate metabolism and phosphorylated uracil nucleotides were largely absent in D39 Δ*proABC*, indicating these metabolic effects may underpin the differences in serum growth rates between these strains.

In conclusion, we have demonstrated that Fhs and therefore one-carbon metabolism have multiple effects on the metabolic pathways required for *S. pneumoniae* growth in human serum or CSF and therefore virulence, data that are potentially relevant for multiple other pathogens that contain Fhs. In addition, we have identified a strain-dependent role for proline biosynthesis for *S. pneumoniae* virulence, showing that bacterial virulence genes can be divided into universal and core-strain-specific categories reflecting differences between strains in their growth requirements under physiological conditions. These differences in metabolic function could also be one mechanism why different strains of *S. pneumoniae* (and other pathogens) vary in their virulence potential.

## MATERIALS AND METHODS

### Strains and growth conditions

Bacteria were cultured in Todd-Hewitt broth (Sigma) supplemented with 0.5% yeast extract (Sigma) in 5% CO_2_ at 37°C or in Columbia agar supplemented with 5% horse blood (CBA) (Oxoid). Bacteria were stored as 0.5 mL single-use aliquots in THY broth at −80°C with 15% glycerol (OD_595_ 0.4–0.5). Plasmids and mutant strains were selected using spectinomycin (Spec) 150 µg/mL or kanamycin (Kan) 250 µg/mL. *S. pneumoniae* growth in THY, CDM, 100% human sera, or cerebrospinal fluid was determined using a TECAN Spark plate reader (5 × 10^6^ CFU/well in 200 µL volume measuring OD_595_). Stress conditions were generated by adding up to 5 mM paraquat (oxidative stress, Sigma-Aldrich), 200 µM ethylene diamine di-o-hydroxyphenylacetic (cation restriction, EDDA), or NaCl (increased osmolarity). When required, media were supplemented with proline, oligopeptides (pro8x PPPPPPPP, AliAPro FNEMQPIVDRQPPPP, AliBPro AIQSEKARKHNPPPP) (54), or purines, adenine, and/or glycine.

### Construction of mutant *S. pneumoniae* strains

Plasmids and primers are described in Table S1. Mutant strains were constructed by overlap extension PCR as described (70), replacing the target gene with Spec or Kan cassette (71–73). Gene deletions were confirmed by PCR and sequencing. Mutation stability was confirmed by multiples rounds of growth in THY without antibiotics then plating onto blood agar plates with and without antibiotics (data not shown). The Δ*fhs* strain was complemented by ectopic insertion of *fhs* using the promoterless integrative plasmid pPEPY (gift from Jan-Willem Veening) (Addgene plasmid # 122633) (49).

### Mouse infection models

Mouse infection experimental procedures were approved by the local ethical review process and performed according to UK national guidelines under the UK Home Office project license PPL70/6510. Outbred CD1 female mice (Charles River Breeders) 4–6 weeks old were infected with *S. pneumoniae* by intraperitoneal injection (5 × 10^6^ CFU in 100 µL, sepsis model), or by intranasal inoculation under isoflurane anesthesia for the pneumonia (1 × 10^7^ CFU bacteria in 50 µL) or nasopharyngeal colonization (1 × 10^7^ CFU bacteria in 10 µL) models. Target organs (nasal washes, lung and spleen homogenates, or blood) were recovered at pre-specified time points, and CFU concentrations calculated by plating serial dilutions onto blood agar plates (14, 74).

### Microscopy

Bacterial cultures grown to OD_595_ 0.2–0.3 were incubated with 1/500 dilution of serotype 6 antiserum (Statens Serum Institute, Denmark), then 1/500 dilution of an anti-rabbit Alexa Fluor 546 antibody (Abcam, UK) (75), and 1/10,000 dilution of DAPI (Biolegend, San Diego, CA, USA). For light microscopy, strain stocks grown in BHI were resuspended in 100% human serum or BHI and cultured for 3 hours, washed, and viewed using a compact confocal laser scanning microscope Zeiss LSM 800 with a 100× objective.

### Flow cytometry C3b, IgG, and phosphocholine binding and neutrophil killing assays

Binding of complement C3b/iC3b or IgG in human sera to live *S. pneumoniae* was detected by flow cytometry as previously described (76). Killing assays using fresh human neutrophils at an MOI of 1:100 and 25% baby rabbit complement (BioRad) were performed as previously described (70), using plating onto blood agar plates to calculate surviving CFU.

### Serum and CSF sources

Human serum from healthy volunteers unvaccinated against *S. pneumoniae* was obtained after obtaining informed consent according to institutional guidelines and stored as single-use aliquots at −80°C. CSF obtained from normal pressure hydrocephalus patients was a kind gift from Diederik van de Beek at UMC, The Netherlands.

### Genome and RNA methods

SP_0931, SP_0932, SP_0933, and SP_1229 conservation among 20,924 pneumococcal genomes in the GPS database was detected using Abricate (version 0.8), using bowite2 version 2.5.3 to calculate coverage (defined as ≥80% identity to the reference genes). For RNAseq, triplicate *S. pneumoniae* OD_595_ of 0.4–0.5 THY cultures was centrifuged and resuspended in 100% fresh human sera or THY for 60 min, before centrifugation and resuspension in RNAprotect (Qiagen). RNA was extracted using Mirvana RNA Kit (Applied biosystems) with an additional lysis step using vigorous shaking with 0.1 mm glass beads (MP Biomedicals), then treated with Turbo DNAse (Applied biosystems). Ribosomal RNA was removed using MICROBExpress (Thermo scientific), and 100 ng was used to construct libraries using the KAPA RNA HyperPrep Kit (Roche Diagnostics, eight amplification cycles), which were single-end sequenced using the NextSeq 500 desktop sequencer (Illumina) and a 75-cycle High-Output Kit (UCL Pathogen Genomics Unit). Raw FASTQ reads were checked by FastQC v0.11.5, Babraham Bioinformatics, UK (77), visualized using multiQC v1.9 (78), trimmed using Trimmomatic v0.39 (79), checked by FastQC and multiQC before mapping to the KEGG annotated *S. pneumoniae* serotype 6B genome sequence (670-6B, accession: CP002176.1) using bowtie2 v2.4.4 with default settings (80). Conversion into BAM files was performed using SAMtools (81). Mapped reads were visualized in the Integrated Genome Browser (82). FeatureCounts v2.0.0 summarized read counts for each annotated feature in multimapping mode (-M) (83). The generated count matrix was imported into R-studio (R v3.4.2), normalized, and differential gene expression analyzed using DESeq2 (84) using log-transformed data for heatmaps and clustering. Differential gene expression was performed on raw counts, using a log_2_ fold change >1.5 and false discovery rate of <0.05 to categorize differentially expressed genes. KEGG pathway enrichment and module analysis were performed in R studio using clusterProfiler (85).

### Lipid mass spectrometry and metabolomics analyses

Strains were grown in THY to an OD_620_ 0.5, centrifuged, and washed twice with PBS before resuspension in human serum at 37°C for 1 hour. Mass spectrometry was performed as described previously (86, 87), with the lipids extracted from washed cells using the Bligh and Dyer method, resuspended in chloroform:methanol (1:1). PtdGro were analyzed using a Shimadzu Prominence Ultra-Fast Liquid Chromatograph (UFLC) attached to a QTrap 4500 operated in the Q1 negative mode and equipped with a Turbo V ion source (Sciex). Samples were injected onto an Acquity UPLC BEH HILIC, 1.7 µm, 2.1 × 150 mm column (Waters) at 45°C with a flow rate of 0.2 mL/min. Solvent A was acetonitrile, and solvent B was 15 mM ammonium formate. The HPLC program was starting solvent mixture 96% A/4% B, 0–2-min isocratic with 4% B; 2–20-min linear gradient to 80% B; 20–23-min isocratic with 80% B; 23–25-min linear gradient to 4% B; 25–30-min isocratic with 4% B. Ion source parameters were ion spray voltage, −4,500 V; curtain gas, 25 psi; temperature, 350°C; ion source gas 1, 40 psi; ion source gas 2, 60 psi; and declustering potential, −40 V. The system was controlled, and data analyzed by the Analyst software (Sciex). For metabolomic analyses, cell pellets were resuspended in 80% methanol containing 0.5 µM warfarin, incubated at −80°C for 1 hour, centrifuged, and the supernatant removed to a new glass tube and dried overnight using a Savant SP1010 SpeedVac. Metabolites were resuspended in 80% methanol and analyzed using UFLC as described above. Samples were injected into an XSelect HSS C18column (2.5 µm pore size, 3.0 by 150 mm) using a flow rate of 0.3 mL/min. Solvent A contained 100 mM ammonium formate (pH 5.0), 2% acetonitrile, and 0.1% t-butanol. Solvent B was composed of 95% acetonitrile, 50 mM ammonium formate (pH 6.3), and 0.1% t-butanol. The HPLC program was starting solvent mixture 0% solvent B, 0–2-min isocratic with 0% solvent B; 2–12-min linear gradient to 5% solvent B; 12–17-min linear gradient to 90% solvent B; 17–25-min isocratic with 90% solvent B; 25–27-min linear gradient to 0% solvent B; 27–30-min isocratic with 0% solvent B. The Sciex QTrap 4500 system was operated in positive (ion spray voltage, 5,500 V; curtain gas pressure, 20 psi; temperature, 400°C; collision gas setting, high; ion source gas 1 pressure, 25 psi; ion source gas 2 pressure, 40 psi) or negative (ion spray voltage 4,500 V; curtain gas pressure, 40 psi; temperature, 500°C; collision gas setting, high; ion source gas 1 pressure, 50 psi; ion source gas two pressure, 50 psi) mode, depending on the metabolite analyzed. The system was controlled by the Analyst software and analyzed with MultiQuant 3.0.2 software (Sciex, Inc.). Metabolites were quantified as normalized abundance to warfarin.

### Statistical analysis

Statistical analyses were performed using GraphPad Prism 8 (GraphPad Software, La Jolla, CA, USA) or R (R v3.4.2). Quantitative results are expressed as median and interquartile range for animal experiments and analyzed using the Kruskal-Wallis non-parametric test. Dunn’s multiple comparisons test was used for post hoc analysis. *P*‐values <0.05 (95% confidence) were considered statistically significant.

## Data Availability

Raw RNAseq data have been deposited in the ArrayExpress database at EMBL-EBI (www.ebi.ac.uk/arrayexpress), under accession number E-MTAB-13289. Raw metabolomics data for the three analyzed strains are available in Data Sets S1 to S3.

## SUPPLEMENTAL MATERIAL

Supplemental figures mbio.01758-24-s0001.pdf Figures S1 to S6.

Legends mbio.01758-24-s0002.docx Legends for the supplemental figures.

Supplemental tables mbio.01758-24-s0003.docx Tables S1 to S4.

Data Set S1 mbio.01758-24-s0004.csv Metabolomics data for 519-43.

Data Set S2 mbio.01758-24-s0005.csv Metabolomics data for BHN418.

Data Set S3 mbio.01758-24-s0006.csv Metabolomics data for DE39.

ASM does not own the copyrights to Supplemental Material that may be linked to, or accessed through, an article. The authors have granted ASM a non-exclusive, world-wide license to publish the Supplemental Material files. Please contact the corresponding author directly for reuse.
