# Supplementary material for: Essential role of proline synthesis and the one-carbon metabolism pathways for systemic virulence of Streptococcus pneumoniae
Source: mBio. 2024 Oct 18;15(11):e01758-24. doi: 10.1128/mbio.01758-24 (PMC11559097; doi:10.1128/mbio.01758-24)

Supplementary Figure 1

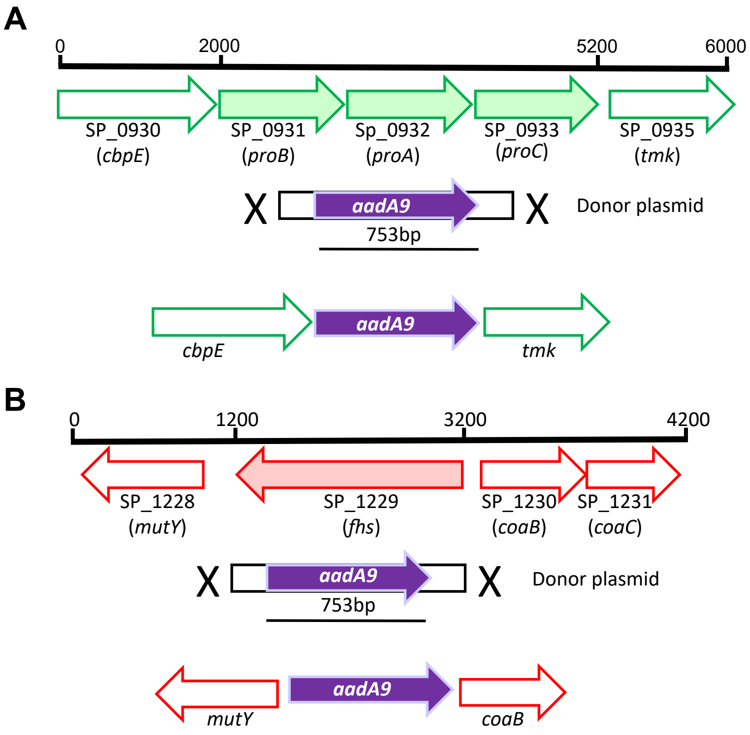

Supplementary Figure 2

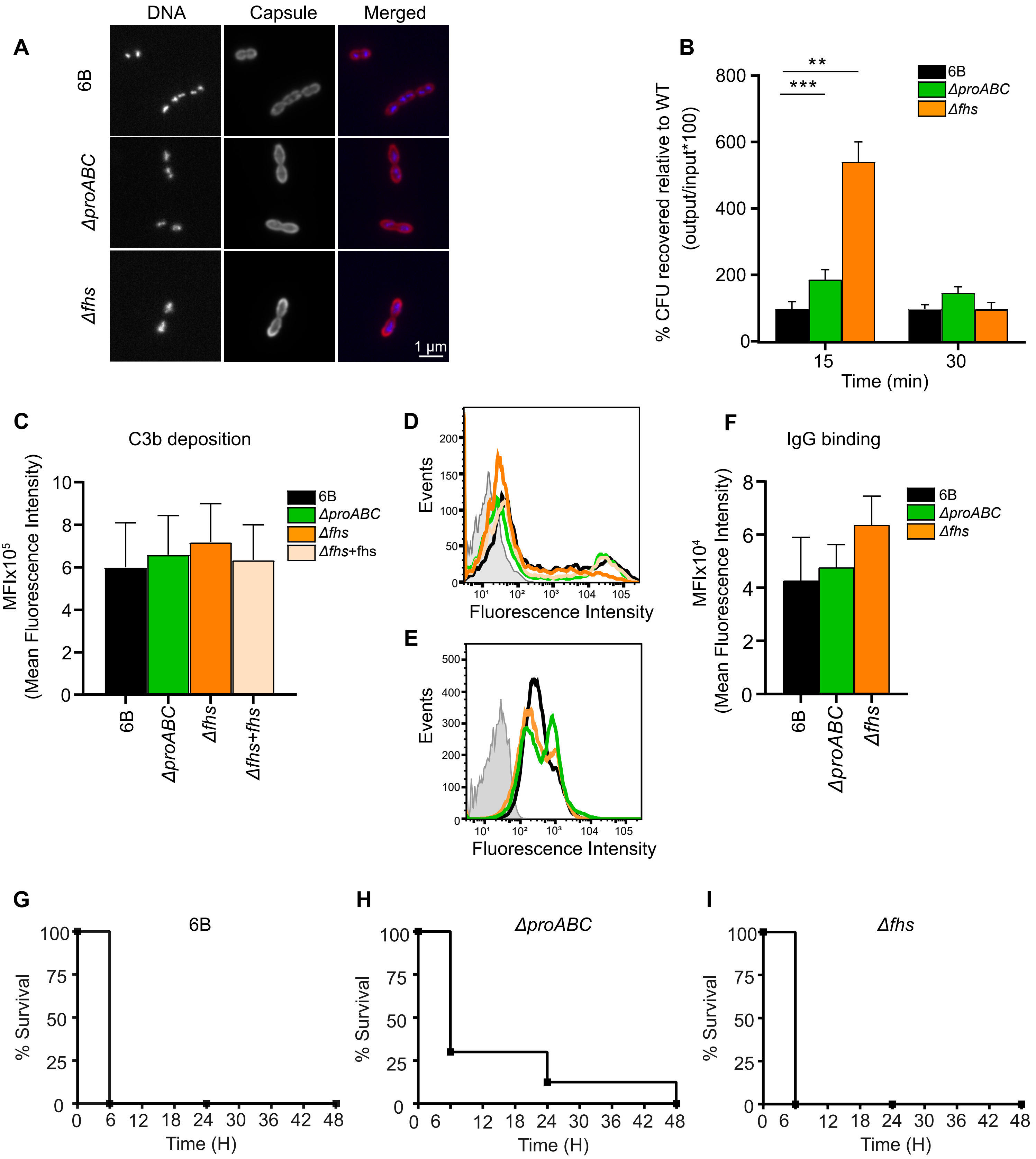

Supplementary Figure 3

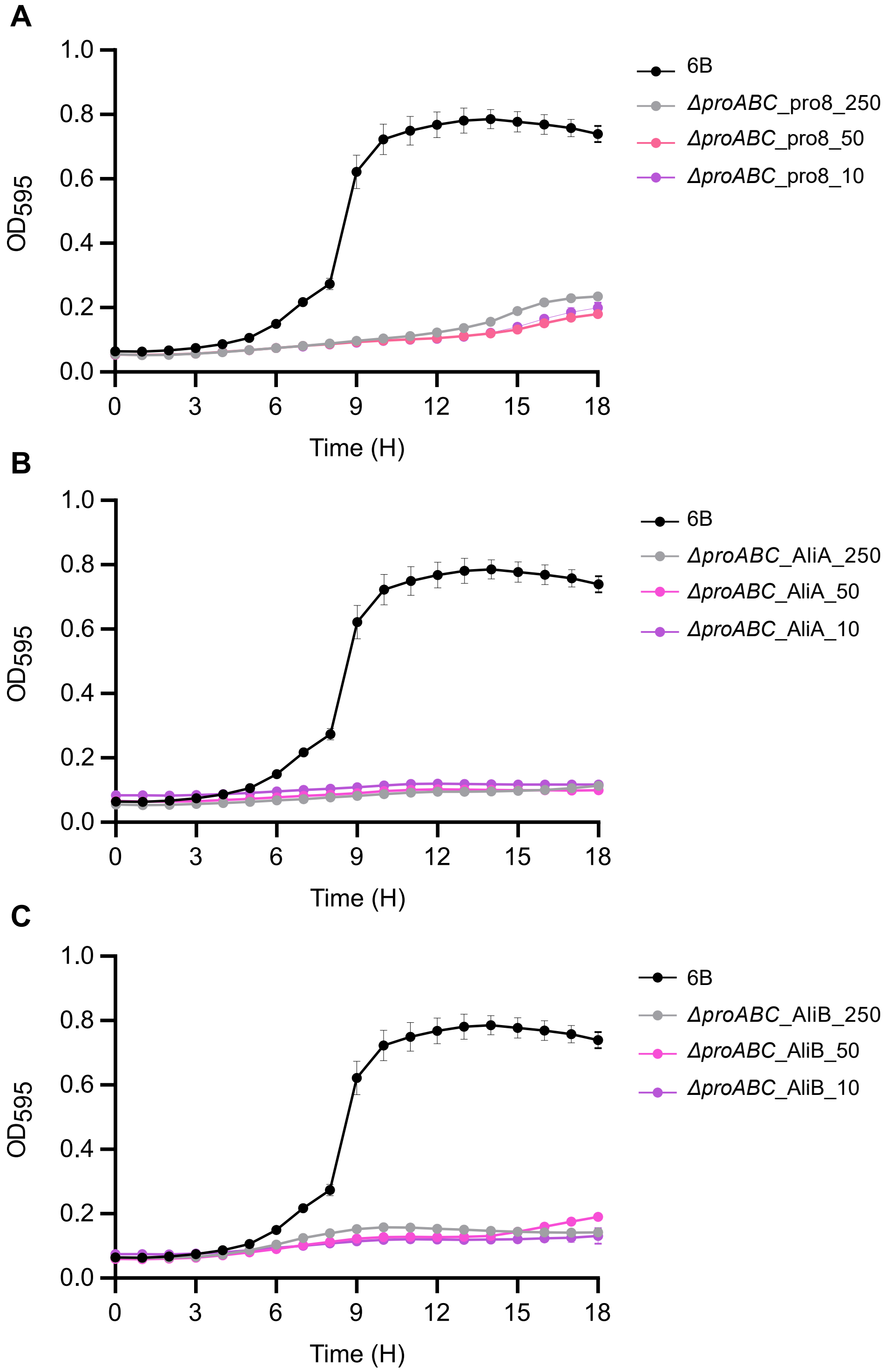

Supplementary Figure 4

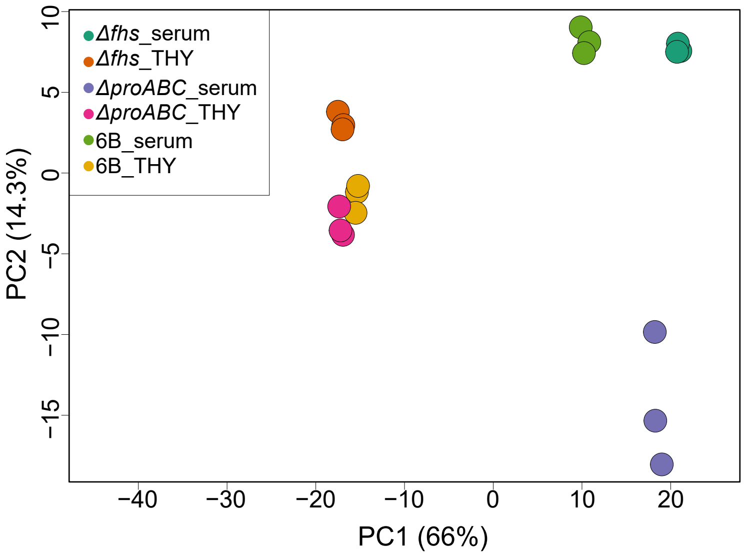

Supplementary Figure 5

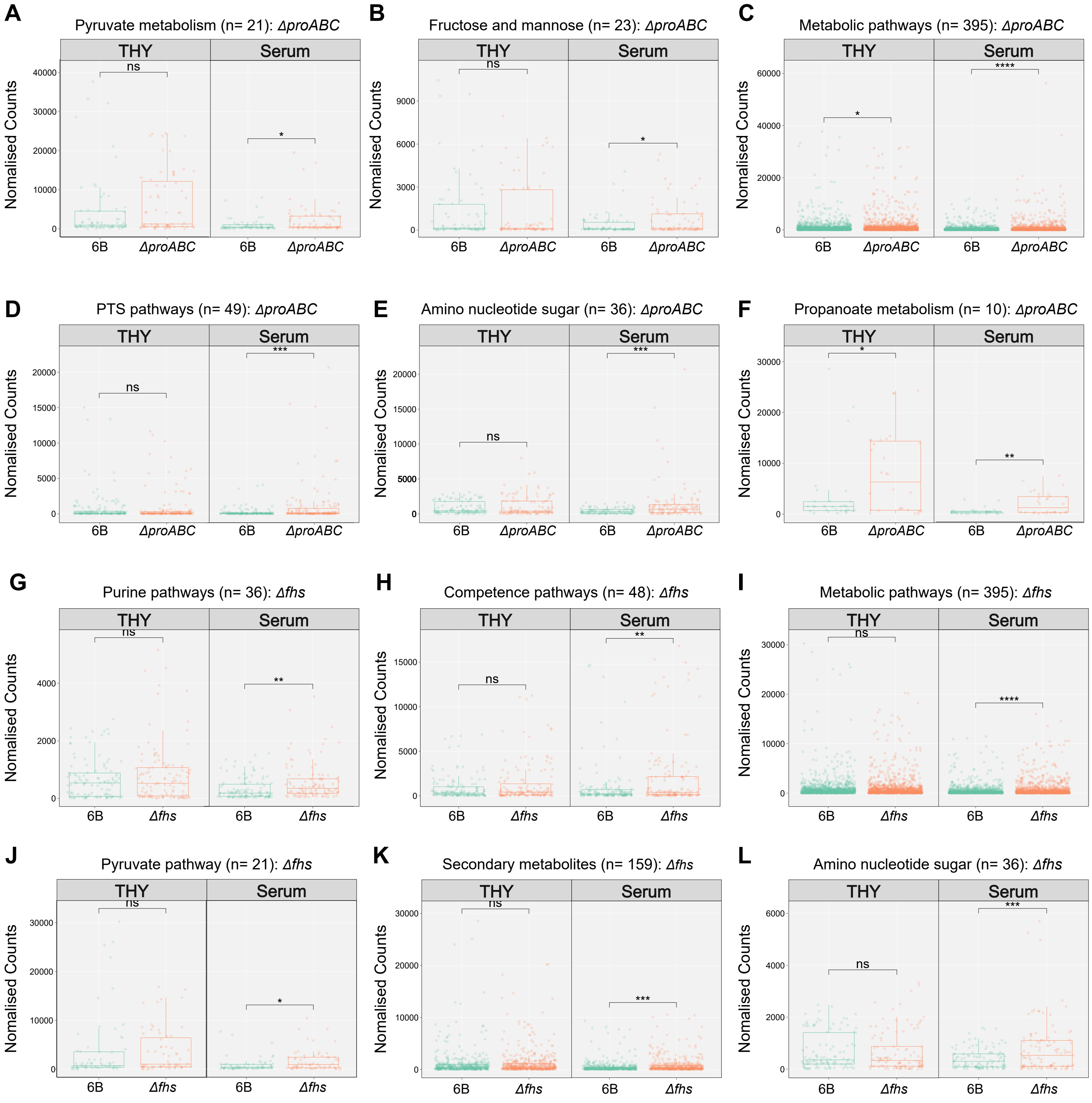

Supplementary Figure 6

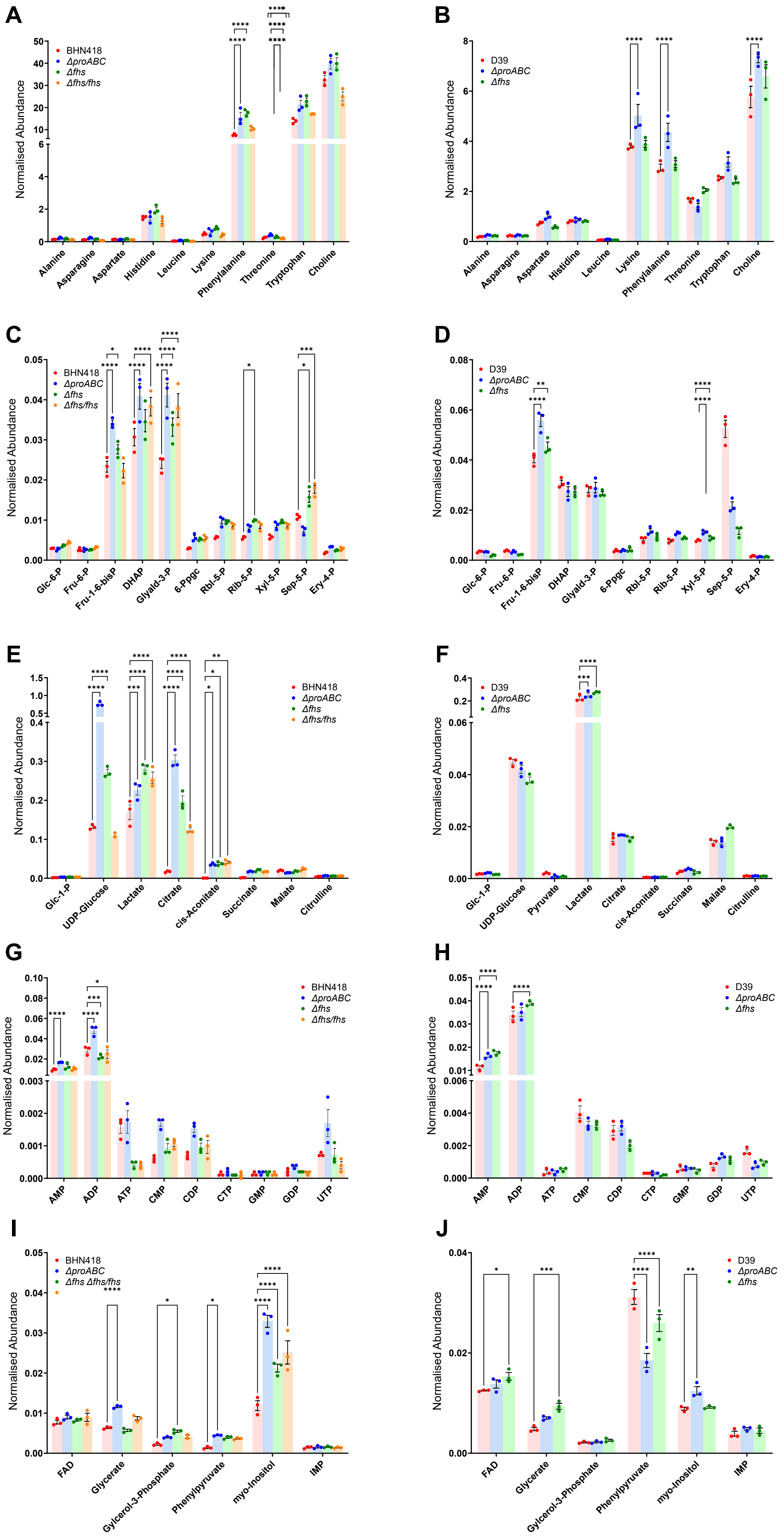

Supplement: Supplemental figures — Figures S1 to S6. [file mbio.01758-24-s0001.pdf]
