# Supplementary material for: Essential role of proline synthesis and the one-carbon metabolism pathways for systemic virulence of Streptococcus pneumoniae
Source: mBio. 2024 Oct 18;15(11):e01758-24. doi: 10.1128/mbio.01758-24 (PMC11559097; doi:10.1128/mbio.01758-24)
Supplement: Legends — for the supplemental figures. [file mbio.01758-24-s0002.docx]

**Supplementary Figures**

**Supplementary Figure 1.** **Organization of the *S. pneumoniae* BHN418 strain *proABC* and *fhs* genetic loci and construction of deletion mutant strains**. (**A**) Schematic of the Sp_0931-0933 locus with the TIGR4 genome gene number and the assigned gene names in parentheses, if available. Arrows indicate transcriptional direction. The putative *proABC* operon is shaded in green colour. In the *ΔproABC* mutant strain the Sp_0931-0933 locus is replaced with an in-frame copy of spectiniomycin (aadA9), shaded purple. (**B**) Schematic of the Sp_1229 locus with the TIGR4 genome gene number and the assigned gene names in parentheses, if available. Arrows indicate transcriptional direction, and *fhs* is shaded in pink. In the *Δfhs* mutant strain Sp_1229 is replaced with an in-frame copy of spectiniomycin (aadA9), shaded purple.

**Supplementary Figure 2**. **In vitro analyses of the effects of deletion of *fhs* and *proABC* on *S. pneumoniae* susceptibility to host immunity.** (**A**) Fluorescent microscopy of wild-type and mutant strains following incubation with 4′,6-diamidino-2-phenylindole (DAPI) (binds to DNA to identify bacterial cells, first column of panels) or antiserum to the 6B capsule labelled with Alexa fluor 546 (panels in second column of panels). The third column of panels represents the merged picture derived from the first two columns. The scale bar (bottom right) represents 1 µm. (**B**) Bacterial survival in a neutrophil killing assay (multiplicity of infection 1 bacterium:100 neutrophils) represented as %CFU recovered after 15 or 30 min incubation compared to the inoculum. (**C**) Mean fluorescence Intensity (MFI) of C3b deposition measured using a flow cytometry assay on 6B, *Δfhs*, *Δfhs+fhs* and *ΔproABC* in 25% of human serum. (**D**) Example of flow cytometry histogram for C3B deposition. Grey shading indicates the results for bacteria incubated in PBS alone. (**E**) Example of flow cytometry histogram for IgG binding. Grey shading indicates the results for bacteria incubated in PBS alone. (**F**) MFI of IgG binding to the 6B strain, *Δfhs* and *ΔproABC* in 25% of human serum measured using a flow cytometry assay. (**G to I**) *S. pneumoniae* *C. elegans* killing assay for nematodes fed *S. pneumonaie* wild-type 6B, ∆*proABC* or *∆fhs* strains, showing some degree of delayed killing for the *∆proABC* strain. Similar results were obtained in 3 independent experiments.

**Supplementary Figure 3**. **Peptide supplementation of *∆proABC* strain growth.** (**A**, **B**, **C**) Growth of wild-type and *ΔproABC* mutant strain in CDM complemented by (**A**) pro8x peptide (PPPPPPPP), (**B**) AliA (FNEMQPIVDRQPPPP) or (**C**) AliB (AIQSEKARKHNPPPP) at three different concentrtions; 250, 50 and 10 ug ml^-1^. Growth was assessed by measuring OD_595_ every 30 min for a 24 hours period of culture at 37˚C and 5% CO_2_ using a plate reader to measure OD_595_.

**Supplementary Figure 4**. **Principal component analysis of normalised and transformed transcripts for 6B, *∆proABC* and *∆fhs* strains in human serum and THY.**  Coloured symbiols represent RNAseq data for a specific strain (performed in triplicate).

**Supplementary Figure 5**. **Pathway enrichment analysis for growth of mutants in THY or human serum, compared to wild-type.** (**A-L**) Overall expression of all genes within six selected metabolic pathways chosen from the pathways enrichment were compared for *∆proABC* (**A-F**) and *∆fhs* (**G-L**) strains using both the THY and serum RNAseq data.

**Supplementary Figure 6.** **Metabolomics analyses for** **intracellular levels of additional metabolites for BHN418 and D39 wild-type strains compared to *∆proABC*, *∆fhs* and *∆fhs+fhs*.** Intracellular levels of (**A** and **B**) amino acids, (**C** to **H**) tricarboxylic acid cycle or carbohydrate metabolism products, or (**I** and **J)** nucleotides additional to those represented in Figure 7. Results are expressed as normalised abundance. Asterisks indicate significant differences between the wild-type and the mutant strains by using a 2-way ANOVA ( * *p* < 0.05; ** *p* < 0.01; *** *p* < 0.001; **** *p* < 0.0001).
